# Small theropod-dominated dinosaur footprint assemblages in the Middle Jurassic Valtos Sandstone and Kilmaluag Formations on the Isle of Skye, Scotland

**DOI:** 10.1098/rsos.251016

**Published:** 2025-09-17

**Authors:** Tone Blakesley, Paige E. dePolo, Dugald A. Ross, Neil D. L. Clark, Stephen L. Brusatte

**Affiliations:** ^1^ School of GeoSciences, University of Edinburgh, Edinburgh, UK; ^2^ School of Biological and Environmental Sciences, Liverpool John Moores University, Liverpool, UK; ^3^ Department of Curation, Staffin Museum, Staffin, UK; ^4^ Hunterian Museum & Art Gallery, University of Glasgow, Glasgow, UK

**Keywords:** dinosaur footprints, dinosaur tracks, Middle Jurassic, Isle of Skye, Valtos Sandstone Formation, Kilmaluag Formation, theropod footprints, theropod tracks, Bathonian

## Abstract

Middle Jurassic deposits on the Isle of Skye, Scotland, are improving our understanding of the distribution, palaeoenvironmental preferences and behaviour of theropod dinosaurs from a time when the global fossil record is sparse. Here, we describe and classify 185 Bathonian-aged *ex situ* dinosaur tracks from Skye’s Trotternish Peninsula—many described for the first time and imaged using photogrammetric techniques—into four morphotypes within a new Hebridean series. In the freshwater, closed-lagoonal Kilmaluag Formation at Lùb Score, smaller morphotypes are more abundant than larger equivalents in the freshwater–brackish fluviodeltaic Valtos Sandstone Formation at Valtos. Although assessable outcrops of track-bearing horizons are limited, we infer that the proximity to, or suitability of, specific palaeoenvironments for different-sized trackmakers may influence assemblage composition. Scarce surfaces with multiple tracks indicate potential trackmaker behaviours in respective palaeoenvironments, including foraging at Valtos and post-hatchling care at Lùb Score. The tracks most likely represent traces of a large megalosaurid and multiple smaller-bodied basal coelurosaurian or non-coelurosaurian (e.g. Ceratosauria, Megalosauroidea, Allosauroidea) theropods. The documentation of these trackmakers and their behaviours further enriches our understanding of dinosaur faunas during this poorly known time.

## 1. Introduction

Our understanding of the evolutionary radiation of a variety of dinosaur clades, which include herbivorous ornithischians, long-necked eusauropods/neosauropods and carnivorous theropods (such as allosauroids and coelurosaurs), during the Middle Jurassic (*ca* 174−164 Ma) [1–4] is constrained by a sparse global fossil record [5].

New, and previously known, dinosaur occurrences from the Late Bajocian-Bathonian aged Great Estuarine Group (*ca* 170−166 Ma) on the Isle of Skye, Scotland, continue to inform on the evolutionary relationships of Middle Jurassic dinosaurs. Some of these discoveries are body fossils and include a thyreophoran ulna and radius; several sauropod teeth and vertebrae, and a humerus; a variety of theropod teeth and a middle caudal vertebra; and a fragmentary specimen with associated bones that might belong to an ornithischian, perhaps an ornithopod [6–13]. Such specimens, however, are very rare and nearly all show signs of transport, which is to be expected in the fluviodeltaic setting they are found in, but renders uncertain the actual environments and habitats these dinosaurs lived in.

Dinosaur tracks, in contrast, are much more abundant on Skye and provide greater insight into palaeoenvironmental preference and behaviour. These have been scientifically documented throughout the Great Estuarine Group on Skye since 1982 [14–16] and include several *in situ* tracksites. These collectively provide evidence of a varied assemblage of sauropod, theropod, thyreophoran and putative ornithopod trackmakers [17–22]. The most abundant trackmakers are theropods [17,18,20–23]. Theropod tracks occur throughout the Great Estuarine Group and are well represented by a wealth of *ex situ* tracks from the Valtos Sandstone Formation at Valtos, and Kilmaluag Formation at Lùb Score on the Trotternish Peninsula [23,24]. Some previously described tracks are tiny to medium-sized and represent the smallest dinosaur trackmakers in the Great Estuarine Group [24,25]. Landmark analysis conducted by [25] on footprints from Valtos and Lùb Score revealed that small (<15 cm length) tracks were indistinguishable between the two localities. Despite this, there has yet to be a systematic and well-illustrated survey of the theropods from these formations.

In this study, we catalogue and comprehensively describe 185 theropod tracks from Valtos and Lùb Score. We group these tracks into a newly devised Hebridean morphotype series based on distinct metrics and morphologies to improve the characterization and classification of tracks found in the Inner Hebrides; and encourage the orderly classification of tracks discovered in the future. This is crucial to understanding assemblage structure and how small theropods interacted with palaeoenvironments during the Middle Jurassic. Notably, Middle Jurassic assemblages with theropod tracks, although present elsewhere, are often infrequent and/or have not been thoroughly described using imaging methods like photogrammetry [26–29]. Some assemblages only or predominantly feature mid-sized tracks, often solely attributed to megalosaurids [30,31], rather than the range of track sizes seen on Skye. Therefore, our detailed description of the Skye tracks provides a key window into the variety and behaviour of meat-eating dinosaurs during the poorly known Middle Jurassic.

### 1.1. Previous work and track discoveries

In 1964/1965, one of us, D.A.R., a schoolboy at the time, discovered a set of eight theropod tracks in a large slab composed of upper Valtos Sandstone Formation (later catalogued as SM.1976.2002.008) on a grassy bank below cliffs north of Kilt Rock from which it had fallen. This finding occurred almost a decade prior to the earliest recorded assemblage of dinosaur bones in May 1973 by Savage and colleagues [13], and approximately 18 years prior to the discovery of an isolated large tridactyl track by Andrews in June 1982—later described by [14]. Thus, D.A.R.’s recollection provides earlier recognition of dinosaurs in Scotland. SM.1976.2002.008 remained on the shoreline for approximately 40 years due to its large size. During this time, D.A.R. monitored the slab to ensure its safety. In 2002, a storm caused part of the grassy bank to collapse onto the shore, with the track-bearing slab vulnerable. Shortly afterwards, D.A.R. organized its recovery with the help of local people. The slab was rolled on planks onto an ex-army assault craft and transported to the Staffin Museum via Staffin slipway. A second, larger track was subsequently discovered upon turning the slab over. Today, SM.1976.2002.008 is exhibited in the Staffin Museum alongside many more tracks found in subsequent years by some of us (D.A.R., T.B., N.D.L.C., Paul Booth (P.B.), Cathy Booth (C.B))—many are described for the first time by this study.

The earliest described tracks from the Valtos Sandstone Formation were recorded in an *ex situ* block (GLAHM 101273/1-38) north of Port Earlish by one of us, N.D.L.C., in January 1996 [32]. The nine tracks constituted two partial trackways respectively referred to *Eubrontes* and *Grallator* [23]. Further tracks from Valtos were discovered at Dun Dearg by D.A.R., N.D.L.C., and P.B. in 2002 and later included in analyses by [25]. In 2016, N.D.L.C. found and described an *ex situ* isolated sauropod track—the only presently known example from the Valtos Sandstone Formation on Skye [11]. Subsequent *ex situ* tracks have been documented intermittently between Port Earlish and a rockfall ~600 m north of Kilt Rock by D.A.R. and T.B. Many of these tracks are reposited in the Staffin Museum and The Hunterian, Glasgow, whilst some remain on the shore because their dimensions and location make them impractical or dangerous to move. A notable recently discovered set of tracks include two large track-rich surfaces found at Carraig Mhòr by T.B in August 2023—one contains at least 36 tracks (specimen VA10).

The earliest described tracks from the Kilmaluag Formation were discovered at Lùb Score in *ex situ* slabs in late 2002 by D.A.R. and P.B.—later described by [24]. Specimens include SM 1976.2002.007—a track-rich slab which at the time was recognized to contain 23 tiny to small-sized tridactyl footprints (<14 cm) (28 are now recognized) bearing the same direction as a single 22 cm long ‘*Eubrontes*’ track [24]. The slab is currently exhibited in the Staffin Museum, while a cast (GLAHM 114912) is held by The Hunterian. The shared directional associations of these trackmakers may represent gregarious, family behaviour akin to post-hatchling care [24]. A smaller *ex situ* slab (GLAHM 114913), also found in 2002, contains three tridactyl tracks. One of them, GLAHM 114913/1, measured 1.78 cm in length and was impressed into the centre of an 8.9 cm long tridactyl track. Further *ex situ* tracks from Lùb Score have been recorded by D.A.R. and T.B. and are either reposited at the Staffin Museum and The Hunterian or remain on the shore. The most recently discovered track-bearing surface reposited with the Staffin Museum was found by T.B. in September 2024 and contains at least 32 tracks (SM.1976.2024.003).

T.B. described many of the tracks featured in our present study for his MScR dissertation at the University of Edinburgh [33].

### 1.2. Geological context

The Great Estuarine Group (*ca* 170−166 Ma) is composed of a series of paralic sedimentary formations deposited in dynamic freshwater and marine-influenced delta and lagoonal systems [34–36]. During this time, Skye was part of a series of landmasses made up of the Hebrides platform and Scottish landmass, which were collectively separated from neighbouring British landmasses by shallow seas [37,38]. The landmasses arose due to intermittent uplift and declining eustatic sea levels [36,39]. The sediments composing Valtos and Lùb Score were deposited in the Sea of the Hebrides basin, situated between two hypothetical local land bodies presently occupied by the Outer Hebrides (Hebrides platform) and Cullin Mountains (Mid-Skye high) [36,38,40]. In the Bathonian, Valtos was situated approximately 42.7° N, while Lùb Score was situated approximately 43.4° N [41].

#### 1.2.1. Stratigraphic context—Valtos

**Figure 1.**
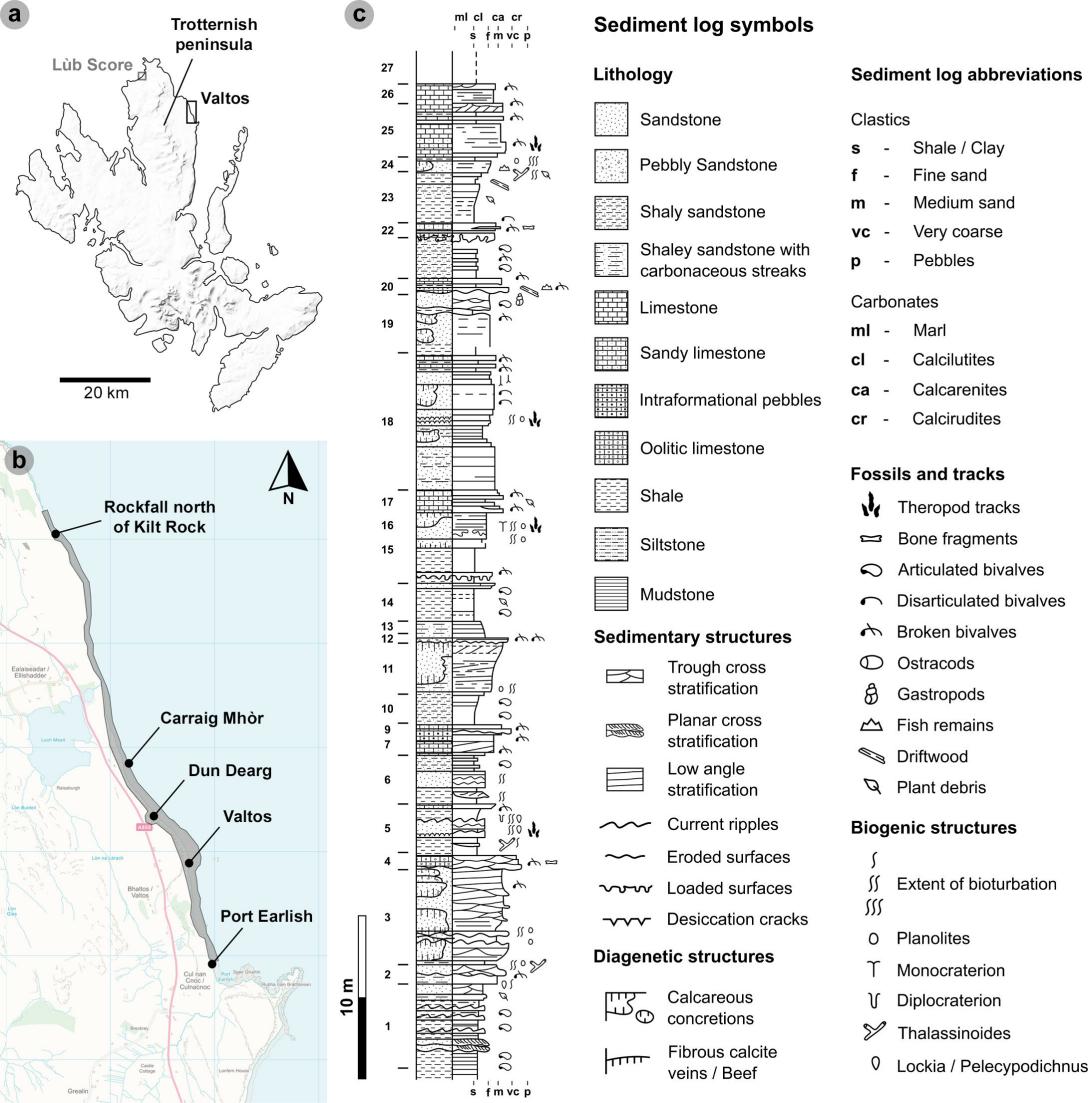
Geographical and geological context of the Valtos tracksite. (a) Valtos is situated on the northeast coast of the Trotternish peninsula. MiniScale^®^, Scale 1:1 000 000, Tiles: GB, updated: 20 November 2023, Ordnance Survey (GB), using: EDINA Digimap Ordnance Survey Service, downloaded: April 2024. Contains public sector information licensed under the Open Government Licence v3.0. Available at: https://www.nationalarchives.gov.uk/doc/open-government-licence/version/3. (b) Tracks have been recorded from the following sublocalities: Port Earlish, Valtos, Dun Dearg, Carraig Mhòr, and a rockfall north of Kilt Rock. OS VectorMap^TM^ District [TIFF geospatial data], Scale 1:25 000, Tiles: ng46_clipped,ng56_clipped, updated: 24 October 2023, Ordnance Survey (GB), using: EDINA Digimap Ordnance Survey Service, downloaded: April 2024. Contains public sector information licensed under the Open Government Licence v3.0. Available at: https://www.nationalarchives.gov.uk/doc/open-government-licence/version/3. (c) Sediment log of the Lower Valtos Sandstone Formation with theropod tracks highlighted from correspondending beds. The log is adapted from [34] to highlight identifiable track-bearing horizons and newer observations.

The Valtos Sandstone Formation (approx. 168 Ma) represents a series of clastic sediments deposited in a fluviodeltaic, freshwater–brackish ‘tidally influenced shore lagoon complex’, during a period of delta progradation and intermittent subaerial exposure [34, p. 240, 36, 38]. The 110 m thick sequence exposed at Valtos is the type section of the Valtos Sandstone Formation and outcrops between Port Earlish and Breun Phort (figure 1). The section is composed of 51 beds, which are divided into a lower, middle, and upper unit [34].

The lower unit is 48 m thick and dominated by fine- to coarse-grained sandstones with occasional large calcareous concretions, shale, mudstone, and limestone (figure 1) [34]. The lower unit crops out between Port Earlish and Carraig Mhòr. We were able to correlate the oldest track-bearing horizons to bed 5. We observed in the lowermost of these, a well-indurated, well-sorted fine-grained sandstone, with grains between 100 and 210 μm. The sandstone weathers to a brick red/orange colour from pale grey on fresh surfaces. In these horizons, we found tracks on both rippled surfaces and those with desiccation cracks; the latter generally preserves positive relief tracks with sharper digit margins. The horizons were deposited toward the top of the facies B sequence, and we interpret them to represent facies 7a—the ‘desiccation crack facies’ [36]. The facies represent ‘a delta plain lagoon or bay bordered by wave-rippled (flaser-bedded) mudflats cut locally by ephemeral channels’ [36, p. 125]. The desiccated bed 5 horizon is interpreted as washover lobe sands which infilled the lagoon or shore margin and dried after subaerial exposure [36, p. 125].

We determined that the uppermost bed 5 track-bearing horizons consist of a moderately sorted, medium-grained sandy limestone, predominantly composed of carbonate with appreciable clastic input (grains are on average approx. 500 μm). The substrate is grey on freshly exposed surfaces and weathers orange, and contains disarticulated, calcified *Neomiodon* bivalves (*sensu* [42]) and cross-cutting calcite veins diagonal to the bedding plane. The track-bearing surfaces are generally featureless and contain positive relief tracks with broad and round digit margins. The horizon marks the end of bed 5 and the facies B sequence of [36] and is succeeded by shale. The sandy limestone represents facies 5—the ‘*Neomiodon* debris limestone facies’, which records sediment abandonment and the subsidence of local shorelines (brackish transgression of the delta plain) [36].

At Dun Dearg, we identified sharp positive relief tracks across at least two horizons with desiccation cracks within bed 18—an approximately 34 cm thick poorly to moderately sorted, fine-grained sandstone with *Neomiodon* bivalves. Grain sizes are typically 80−150 μm. The lower desiccated horizon features shale intercalations and contains *Planolites*. A rippled horizon, where we observed no tracks, separates the lower track-bearing horizon from a heavily bioturbated horizon with *Planolites*. The bed 18 sandstones represent facies 2 within facies sequence C [36]. The sandstones represent flood derived sand sheets and are part of a shoreline progradation sequence of basic, interdeltaic lagoon shorelines [36]. The succession of desiccated and rippled surfaces indicates periodic fluctuations in emergence.

To the north of Dun Dearg, we recorded a single, partial positive relief track (VA12) in a boulder we correlated to bed 16—a finely laminated, grey coloured fine-grained sandstone. The surface is heavily bioturbated by *Planolites* and *Monocraterion*. Bed 16 represents facies 2—an organically productive, moist lagoonal shoreline substrate [36].

Further north at Carraig Mhòr, we recorded positive relief tracks on the surface of two large *ex situ* blocks composed of a moderately sorted, weakly laminated, medium- to coarse-grained sandy limestone with broken-disarticulated *Neomiodon* bivalves. The sandy limestone is light grey on fresh surfaces (weathering pale brown). The track-bearing surface is generally featureless but may preserve faint ripples. We correlated the horizon to bed 25, which represents facies 5 in the facies sequence C (*sensu* [36]). The sandy limestone reflects a reduced availability of clastic sediment, allowing more carbonate to precipitate [36].

The overlying 27 m thick middle unit is predominantly composed of *Neomiodon* bivalve sparites in grey limestones and shales [36]. The sequence indicates the development of transgressive brackish conditions and an increase in organic productivity [36]. The unit is exposed from the Dun Dearg rockfall to Breun Phort and has yet to yield tracks.

Increased sediment transport is indicated in the 46 m thick upper sandstone-dominated unit, gradually exposed north from Dun Dearg. Clastic sediments include ‘medium to very coarse trough and planar cross stratified sandstones’, which dominate the sequence until the Duntulm Formation [34, p. 241]. Tracks can be preserved in positive relief on medium- to coarse-grained sandstone surfaces with desiccation cracks. The grains are well sorted and range between 180 and 400 μm (mean = 340 μm). Freshly exposed surfaces are grey but weather to a dark orange/brown colour and occasionally feature disarticulated *Neomiodon* bivalves. Tracks from this unit have typically been found in loose blocks and could not be correlated to a precise bed due to unsafe site access.

#### 1.2.2. Stratigraphic context—Lùb Score

**Figure 2.**
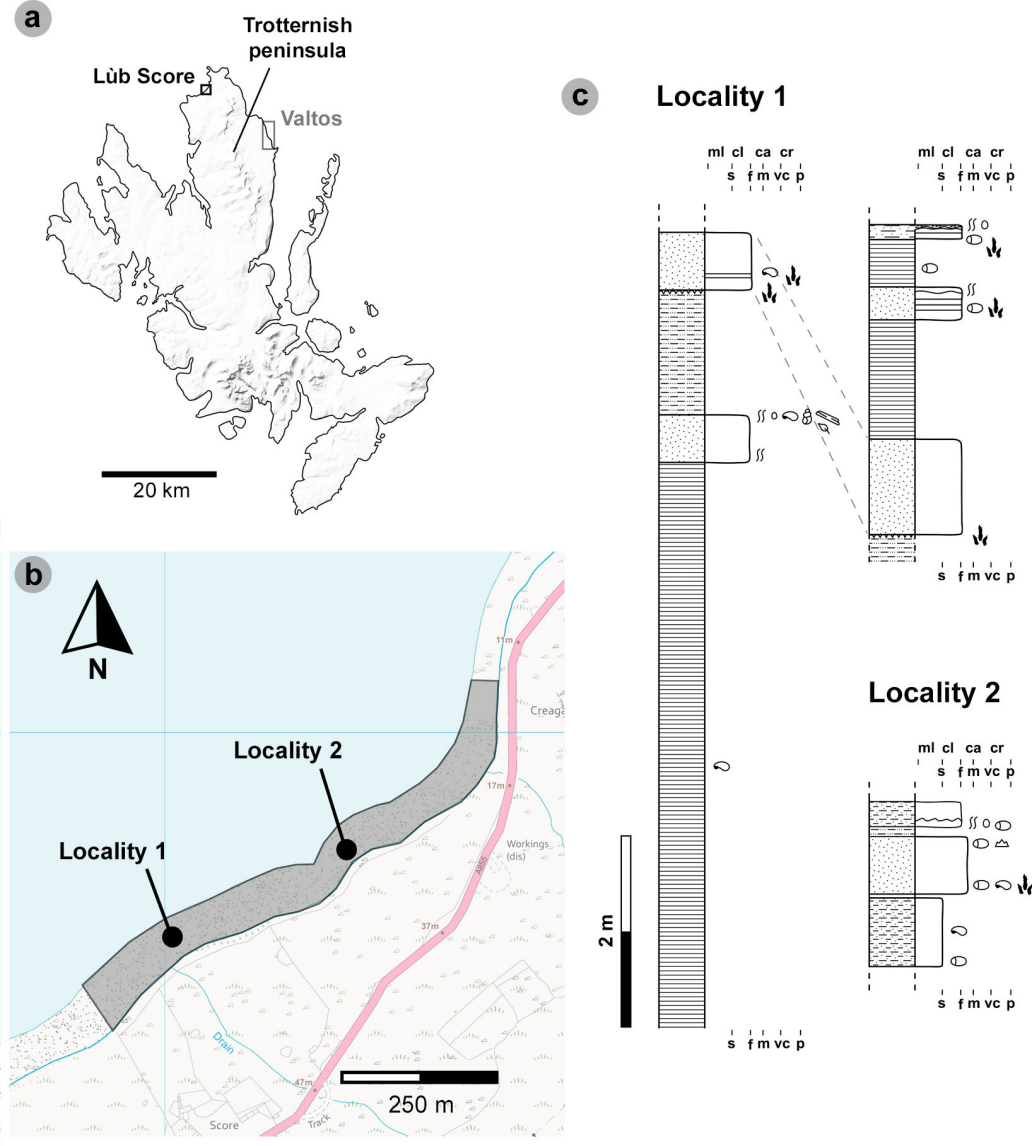
Geographical and geological context of the Lùb Score tracksite. (a) Lùb Score is situated on the northeast coast of the Trotternish peninsula. MiniScale^®^, Scale 1:1 000 000, Tiles: GB, updated: 20 November 2023, Ordnance Survey (GB), using: EDINA Digimap Ordnance Survey Service, downloaded: April 2024. Contains public sector information licensed under the Open Government Licence v3.0. Available at: https://www.nationalarchives.gov.uk/doc/open-government-licence/version/3. (b) Tracks have been recorded from the following sublocalities: locality 1 and locality 2. Note that the locality 1 sediment log is split in two and accounts for two exposures. OS VectorMap^®^ Local [TIFF geospatial data], Scale 1:10 000, Tiles: ng37se_clipped,ng47sw_clipped, updated: 1 January 2024, Ordnance Survey (GB), using: EDINA Digimap Ordnance Survey Service, downloaded: April 2024. Contains public sector information licensed under the Open Government Licence v3.0. Available at: https://www.nationalarchives.gov.uk/doc/open-government-licence/version/3. (c) Sediment logs of the sublocalities at Lùb Score with theropod tracks highlighted from correspondent beds. The logs are adapted and expanded from [24] to highlight newer observations.

The late Bathonian (approx. 166 Ma) clastic facies of the Kilmaluag Formation exposed at Lùb Score represent a low salinity, closed lagoon margin susceptible to drought and sediment influxes [35,43]. Although initially logged by [24], it remains uncertain which part of the Kilmaluag Formation the track-bearing Lùb Score sediments correlate to, as outcrops across Trotternish exhibit considerable lateral variation [34,35]. A nearby outcrop, situated at a higher elevation above the Lùb Score tracksites on the Lon Ostatoin River, was tentatively correlated to the upper part of the formation [35]. However, we are unable to place this outcrop into stratigraphic relationship with the track-bearing beds on the shore. Acknowledging this uncertainty, we identified several track-bearing beds across two intertidal sublocalities at Lùb Score (figure 2). The units exposed in each are relatively unique and can exhibit localized lateral variation.

Locality 1 exposes multiple track-bearing horizons, the lowermost is an ostracod-rich, grey-white coloured, laminated siltstone with desiccation cracks [24]. Tracks from this horizon are best preserved as positive relief casts at the base of an overlying approximately 60 cm thick bed of moderately to well-indurated, well-sorted, fine- to medium-grained sandstone (grain size 100−250 μm), which infilled the siltstone. The sandstone is pale grey in colour on freshly exposed surfaces (weathering brown). At least two further track-bearing horizons exist within the sandstone. This includes a rippled horizon with low to moderate invertebrate bioturbation, which appears approximately 14 cm above the lower contact with the siltstone and contains tiny to small-sized tridactyl tracks between 1.8 and 12.5 cm in length [24]. A second horizon with worn tracks exists directly below a horizon of *Pleuromya* bivalves and is approximately 30 cm above the base of the sandstone (seen in specimen SB02).

In the northeast edge of locality 1, we record two further track-bearing horizons that are higher in the stratigraphy. They are separated from an approximately 100 cm thick sandstone (a lateral variation of the approx. 60 cm thick sandstone with worn positive relief tracks at its base) by a grey mudstone (up to 125 cm thick). The lowermost horizon is approximately 5 cm above the base of an approximately 40 cm thick, well-laminated, fine-grained sandstone. The grains are well to moderately sorted—between 75 and 250 μm (mean = 118 μm). The sandstone contains ostracods and weathers brown (grey on fresh surfaces). The track-bearing horizon lacks desiccation cracks and contains shallowly positive relief tracks with broad digit margins—which may indicate moist, shallowly submerged registration conditions (*sensu* [44]). Similar tracks are recorded in a horizon at the base of an overlying approximately 15 cm thick, organic-rich, well-sorted fine-grained sandstone. This horizon is distinguished with darker streaked laminations. The two sandstones are separated by a well-laminated, light grey, approximately 59 cm thick mudstone.

Locality 2 is approximately 315 m northeast of locality 1, with *ex situ* tracks believed to originate from ‘a [fine- to medium-grained] sandstone containing darker organic laminae’ [24, p. 95]. The white-laminated sandstone bed in question is approximately 19 cm thick and rests above a 4 cm light brown mudstone. The sandstone is thought by [24] to be the lateral equivalent of the lowermost track-bearing sandstone at locality 1. The surfaces of *ex situ* sandstone track blocks, which we subsequently found at locality 2 are varied and can feature ripples or bioturbation—likely implying that multiple track-bearing horizons exist. Due to poor exposures at locality 2, these additional track-bearing horizons could not be confidently located within the known sequence.

In between the sublocalities, we record wave-worn boulders of poorly consolidated, fine-grained sandstone with unevenly eroded cross-sections of tracks. The boulders are common across Lùb Score and are not correlated with the *in situ* stratigraphy.

### 1.3. Sequence biotas

The Valtos biota includes invertebrate ichnotaxa such as *Thalassinoides*, *Monocraterion*, *Diplocraterion*, *Planolites* and *Lockia* [34]. *Neomiodon* bivalves (commonly observed in track-bearing horizons), *Viviparus* gastropods, coniferous driftwood and other plant debris indicate freshwater input [34]. A study by [45] recorded a rich assemblage of spores and pollen florules dominated by pteridophyte spores including *Neoraistrickia gristhorpensis*. *Botryococcus* was also abundantly recorded with the early Bathonian *Lycopodiacidites baculatus* [46]. In addition to this evidence for freshwater/terrestrial input, occasional evidence of fish debris (scales, teeth, jaws, plates) and shark teeth and fin spines indicates brackish conditions and periods of open accessibility to marine palaeoenvironments.

The Kilmaluag Formation biota of Lùb Score differs from the Valtos Sandstone Formation due to its abundance of low salinity ostracods. Some ostracods are recorded in track-bearing horizons, including *Darwinula cicatricosa* and *Theriosynoecum conopium*, and the conchostracan species *Antronestheria kilmaluagensis* in ‘green-grey silty mudstones’ below track-bearing sandstones in locality 1 [24,47–50]. We observed freshwater molluscs, including *Viviparus* gastropods and *Pleuromya* bivalves—the latter found close to track-bearing horizons, and invertebrate ichnotaxa which includes *Planolites*. Occasional fragmentary plant remains occur in sandstones and consist of carbonized driftwood and tiny stems and leaves.

## 2. Material and methods

### 2.1. Specimen cataloguing and description

The Staffin Museum has been collecting and preserving Scottish dinosaur footprints since 2000 and while contextual information for each block was recorded contemporaneously with collection, relatively few footprints were assigned formal collection numbers. To support this study, we catalogued all footprints in the collection. We use the museum’s existing specimen numbering strategy with prefixes to denote: museum (‘SM’ for Staffin Museum); year of establishment (1976); year of specimen acquisition; specimen number for that year; a side letter (if tracks are present on multiple track-bearing horizons on a block); and track number (if multiple tracks are present). For example, ‘SM.1976.2002.008b-3’ is the third track of the eighth specimen to be catalogued in 2002 and features multiple track-bearing horizons. In our tables, we display shortened specimen numbers from the year of specimen acquisition.

Tracks in large, irremovable *ex situ* blocks on the shore were prefixed with a site code (‘VA’ for Valtos, ‘SB’ for Lùb Score), specimen number and track number (if multiple tracks are present). For example, ‘VA10-3’ is the third track present in specimen 10 at Valtos.

For all tracks included in this study, our observations stated the: specimen number, locality of origin (with sublocalities abbreviated), whether a track was left or right, relief, symmetry, preservation grade (details below), lithology, bed number (if assignable) and general track description (see electronic supplementary material, appendix S1 of table S6). Since positive relief tracks result from infilling a negative relief impression, the siding of these footprints on *ex situ* blocks of the base of beds is reversed (e.g. tracks which appear right footed are catalogued as left tracks). Such tracks are denoted by an asterisk after their left or right designation.

### 2.2. Morphological preservation grading

Tracks assessed by this study were assigned morphological preservation grades, which determine, on an ordinal scale of 0−3, how well preserved a track is [51,52]. Tracks graded towards three feature more clearly defined morphologies. The presence or absence, sharpness and wear of morphology for each track inform their preservation grade. Preservation grades are influenced by digits i−iv and their respective ungual marks, and pads as defined by [53–55]. The grading criteria are outlined in [52, table 1]. Complete theropod tracks with an overall grade ≥1.5 were measured and used to characterize morphotypes in the Hebridean series (see §2.8). Tracks graded as ‘1’ were either used to characterize or referred to a Hebridean morphotype, depending on the extent of surface wear, based on shared measurements or track morphology. Tracks graded <1 were generally too incomplete or poorly defined to characterize under a Hebridean morphotype but may feature morphotype-specific morphologies. Such tracks are listed under Hebridean morphotype subgroup ‘referred material’ (see electronic supplementary material, tables S1−S4).

**Table 1.** Theropod tracks as classified by their length in accordance with [58].

trackmaker	length categories	track length (PL)
theropod	tiny	<10 cm
	small	10 cm ≤ PL < 20 cm
	medium	20 cm ≤ PL < 30 cm
	large	30 cm ≥ PL < 50 cm
	giant	PL ≥ 50 cm

### 2.3. Photogrammetric datasets of tracks

Each track was photographed ≥50 times at three different angles in circular rotation on a 24.2-megapixel resolution Nikon D5600 with a 35 mm lens. Larger surface areas with multiple tracks were photographed by hand at near nadir angles and sequentially overlapped. Photographs were taken outdoors in calm weather in bright, diffuse lighting conditions. Camera parameters included aperture ≥f13, shutter speed ≥1/200 and ISO ≤640. Specimens in The Hunterian, University of Glasgow and Staffin Museum that had to be photographed indoors were taken at an aperture ≥f9, shutter speed ≥1/100 and ISO ≤800 due to dimmer lighting (with a camera flash).

### 2.4. Generation of photogrammetric models

To generate photogrammetric models, photographs were imported into Agisoft Metashape (V. 1.8.0, Build 13794, 64 bit, 2023) installed on two workstations based in the University of Edinburgh Airborne Research and Innovation facility. Workstation specs are Athena (i7-7820X8 core (16 with hyper-threading) and 1× NVIDIA GeForce GTX 1080 Ti 11 GB, 128 GB RAM) and Goliath (2× Intel Xeon E5-2640V4/2.4 GHz processor, total 20 cores, 40 threads and 2 ×ASUS GTX1080TI-FE, 256 GB RAM). Once imported, we followed a workflow devised by [21]. The models were scaled either by placing a ruler in the field of view or by measuring the distance between two distinct features on the track-bearing surface. The resolution parameters for dense point cloud, mesh and texture generation were set to the ‘highest’ setting. For sharp texture generation, the source data was set to ‘3D model’ and other fields left on default.

### 2.5. Post-processing photogrammetric models

Meshlab (V. 2022.02, 64 bit) was used to post-process models. Models >2 GB were processed on a University of Edinburgh Geosciences CT laboratory workstation PC (Intel Xeon W-2125 CPU at 4.00 GHz with NVIDIA GeForce GTX 1060 6 GB, 160 GB RAM). A laptop workstation processed models <2 GB (i7-11800H at 2.3 GHz with NVIDIA RTX™️ A3000 (6 GB GDDR6 dedicated) 32 GB RAM). Meshlab was used to remove excess mesh (slab edges, boulders, terrain) to accurately set a plane through the track-bearing surface. Subsequently generated digital representations included textured orthophotos, digital elevation maps (DEMs) and contour maps in Paraview (V. 5.10.1) installed on the laptop workstation. Track outlines were drawn in Inkscape (V. 1.2.2, 64-bit).

### 2.6. Track and trackway measurements

**Figure 3.**
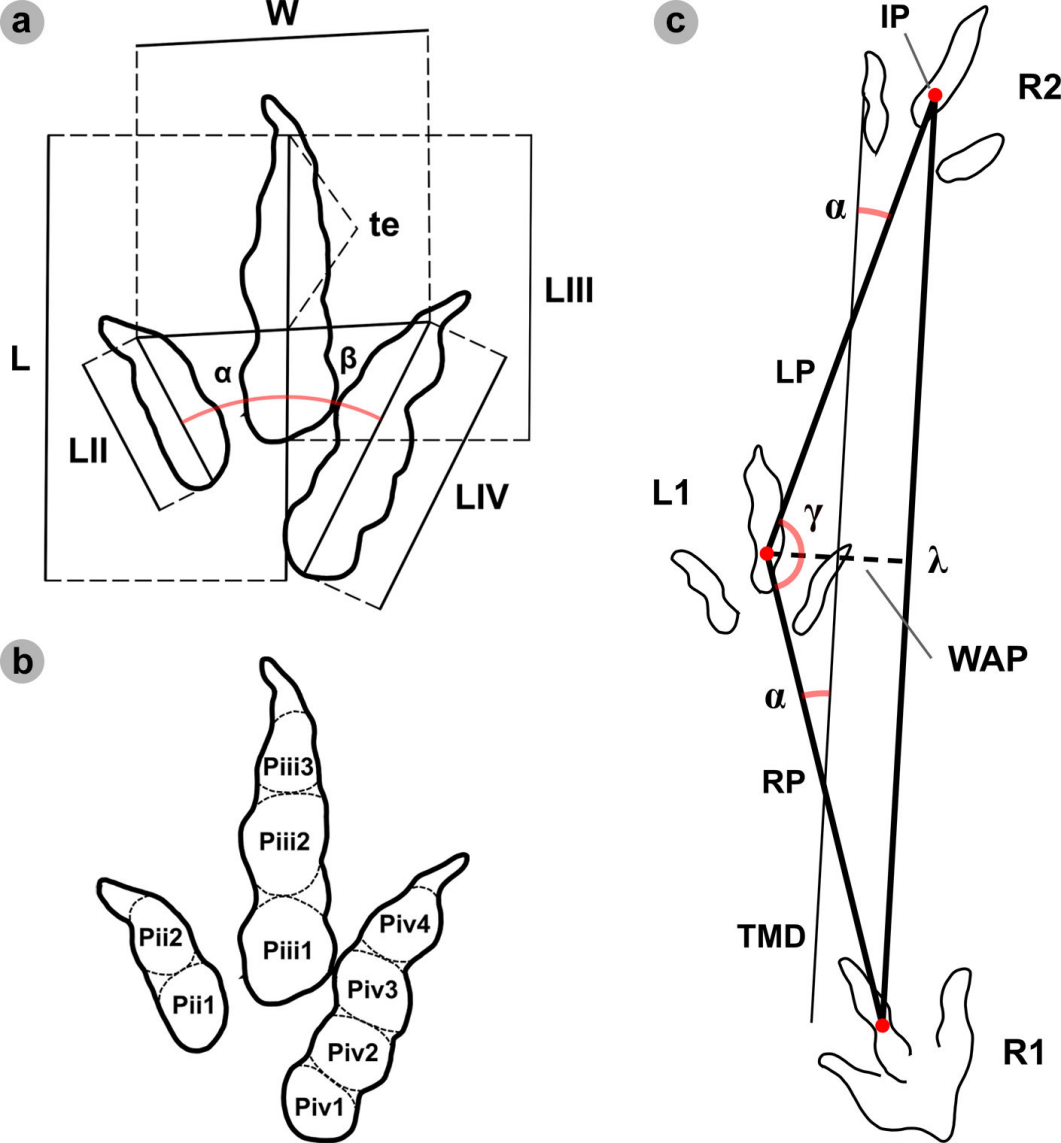
(a) Measurements for theropod tracks included total track length (L), width (W), digit lengths (LII–LIV), digit iii toe extension (te) and interdigital angles between digits ii–iii (*α*) and digits iii–iv (*β*) (which informed a total divarication angle). (b) Theropod track phalangeal pad configuration (2 : 3 : 4 formula) between digits ii–iv (right to left) (*sensu* [57]). (c) Bipedal trackway measurements included: pace (*p*), stride (*λ*), the width of angulation for pes tracks (WAP), pace angulation (*γ*) and angle of rotation (*α*).

For tracks, we measured the total track length (L), width (W), digit lengths (LII–LIV), digit iii toe extension (te) and interdigital angles between digits ii–iii (*α*) and digits iii–iv (*β*) in FIJI ImageJ [56] (figure 3a). These measurements were conducted following [33] in accordance with [58]. Like [58], we excluded ungual marks from length measurements due to their variability between tracks. Our track measurements calculated: length-to-width (l/w) ratios, digit ii/iii and iii/iv ratios; mesaxony (te/W); and digit iii/total track length ratio (iii/L) [59–61]. Tracks were further categorized by length (*sensu* [58]) (table 1). Phalangeal pads were identified following [57] (figure 3b).

For trackways, i.e. sequences with ≥3 tracks [53,54], we measured pace—measured between two intersecting points (IP) of length and width of two consecutive tracks, from alternate feet (RP and LP, respectively); stride (*λ*)—measured between two successive tracks of the same foot; width of the pes angulation pattern (WAP)—the perpendicular trackway width between an IP and stride length line; angle of rotation (*α*)—measured between the forward facing side of a pace line and trackway midline (TML); and pace angulation (*γ*)—the angle of two intersecting pace lines [53,54] (figure 3). The TML was drawn between the midpoints of pace lengths [54]. For track associations, i.e. sequences with two tracks, only pace or stride could be measured.

### 2.7. Trackway-based estimations

Trackmaker hip height (*h*) was estimated by multiplying a track length (FL) by four (equation (2.1)) and informed velocity estimations [62]. Velocity (*V*) was calculated using the gravitational constant (*g* = 9.81), a trackway average stride length (*λ*) and estimated hip height (*h*) (the latter based on the mean trackway track length). An updated version of this equation, devised by [63], is also used (equation (2.3)). Stride length was further divided by an estimated hip height to estimate trackmaker gait (equation (2.4)) [62,64]. Gait values were approximated as walking below 2.0 and running above 2.0 [54,62].


(2.1) h=4×FL,

(2.2) $$V=0.25g^{0.5}\lambda^{1.67}h^{-1.17},$$

(2.3) $$V=0.226g^{0.5}\lambda^{1.67}h^{-1.17},$$

(2.4) $$\text{Gait}=\frac{\lambda }{h}.$$

### 2.8. Morphotype classification

**Figure 4.**
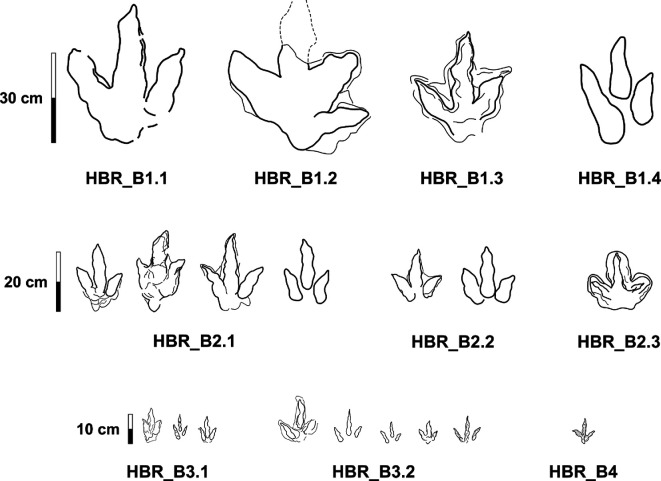
Hebridean morphotypes based on tracks from Valtos and Lùb Score. Some outlines have been mirrored to show left tracks for easier comparison.

Tracks with distinct metrics and morphologies are classified into individual morphotypes, following a scheme that we devised and explain here. These groupings reflect the observed shape and size of the tracks, and remain agnostic about the specific factors which might influence these shapes (e.g. trackmaker anatomy, locomotion or substrate properties). Previously, Romano *et al*. [65] used an existing morphotype series developed for tracks in the Cleveland Basin of England to classify the Hebridean tracks. We reevaluated these morphotypes in the context of describing the Valtos and Lùb Score tracks and found that the Cleveland Basin scheme was not fit for classifying tracks in the Inner Hebrides, given many observed differences between the two track assemblages. Consequently, we devise a new morphotype scheme for classifying the Scottish tracks we are describing: the Hebridean series (figure 4). The scheme expands our earlier site-specific series at Prince Charles’s Point [22] and is intended to be used as a track grouping scheme on the Isle of Skye where dinosaur tracks are regularly being discovered, and potentially on surrounding Hebridean islands with similar geology, where tracks could be discovered in the future. Grouping the Hebridean tracks into these categories is a practical bookkeeping exercise that can help us systematically understand the shape variations between tridactyl tracks within the local assemblages on Skye, and use it as a starting point to begin to make reasonable hypotheses about trackmaker identities and palaeoenvironmental preferences.

Each morphotype features the following prefixes: a three-lettered abbreviation of the basin—i.e. ‘HBR’ for Hebridean; a ‘B’ to denote the trackmakers as bipedal; a morphotype number; and a subgrouping number (when appropriate). For example, morphotype ‘HBR_B2.1’ is the first subgroup of the second bipedal morphotype from the Hebridean Basin. Morphotype subgroups are characterized by distinctive metrics and morphology and represent variation within a morphotype, e.g. l/w ratios, mesaxony, divarication angles and overall track shape. These characteristics were compared with previously described ichnotaxa to suggest a representative ichnotaxon and inform on possible trackmakers.

## 3. Results

### 3.1. Morphotype overview

Three hundred and twenty-five tracks from Valtos and Lùb Score were catalogued and preservation graded. Out of these tracks, 85 were measured and used to characterize one of four morphotypes (including ten morphotype subgroups) (figure 4). A further 100 were referred to respective morphotypes.

**HBR_B1:** Rare and generally characterized by track lengths >30 cm, broad to moderately slender digits (with sub-parallel to parallel-sided margins), elongated ungual marks and predominantly weak-moderate l/w ratios (1.29–1.76) and mesaxony (0.41–0.71). When distinguishable, the digit iv metatarsophalangeal pad is the largest pad. HBR_B1 is split into four subgroups. HBR_B1.1 is >40 cm in length and divaricated between 40° and 60° between digits ii–iv (digit margins are parallel-sided). HBR_B1.2 differs through the presence of a deeply impressed padded hallux and heel and broader digit thickness (digits ii–iv lack pads). HBR_B1.3 is approximately 30 cm in length and is widely divaricated between digits ii–iv (>60°) (digit margins are sub-parallel-sided). HBR_B1.4 is broad digited and mesaxonic with moderate-pronounced l/w ratios (>1.50).

**HBR_B2:** Small to medium-sized tridactyl tracks with moderately slender, spindle-shaped digits. HBR_B2 is divided into two subgroups. HBR_B2.1 is characterized by l/w ratios >1.30 and pronounced mesaxony (generally >0.50) and smaller digit ii–iv divarication angles (<60°). HBR_B2.2 differs with weaker l/w ratios (mostly <1.30) and larger digit ii–iv divarication angles (generally >60°). HBR_B2.3 contrasts with slender inner digit margins enclosed by round and broad digits. HBR_B2 is most abundant in the Valtos Sandstone Formation.

**HBR_B3:** Tiny to small tridactyl tracks with longer than wide, oval-shaped phalangeal pads and gracile digits which taper toward narrow, elongated ungual marks. HBR_B3 is differentiated into two subtle subgroups. HBR_B3.1 possesses pronounced l/w ratios (>1.50) and mesaxonic indices (>0.50)—some larger than HBR_B2.1. HBR_B3.2 features wider digit ii–iv divarication angles (>50°), smaller l/w ratios (generally <1.3) and, when visible, a ‘2 : 3 : 3’ configured phalangeal pad formula. Although both HBR_B3 subgroups can metrically grade into one another, we distinguish them to demonstrate the extreme variability exhibited within this morphotype. HBR_B3 is most abundant in the Kilmaluag Formation.

**HBR_B4:** The tiniest morphotype (track lengths <10 cm) is currently known from Lùb Score and occasionally features a reversed hallux.

### 3.2. HBR_B1.1

**Figure 5.**
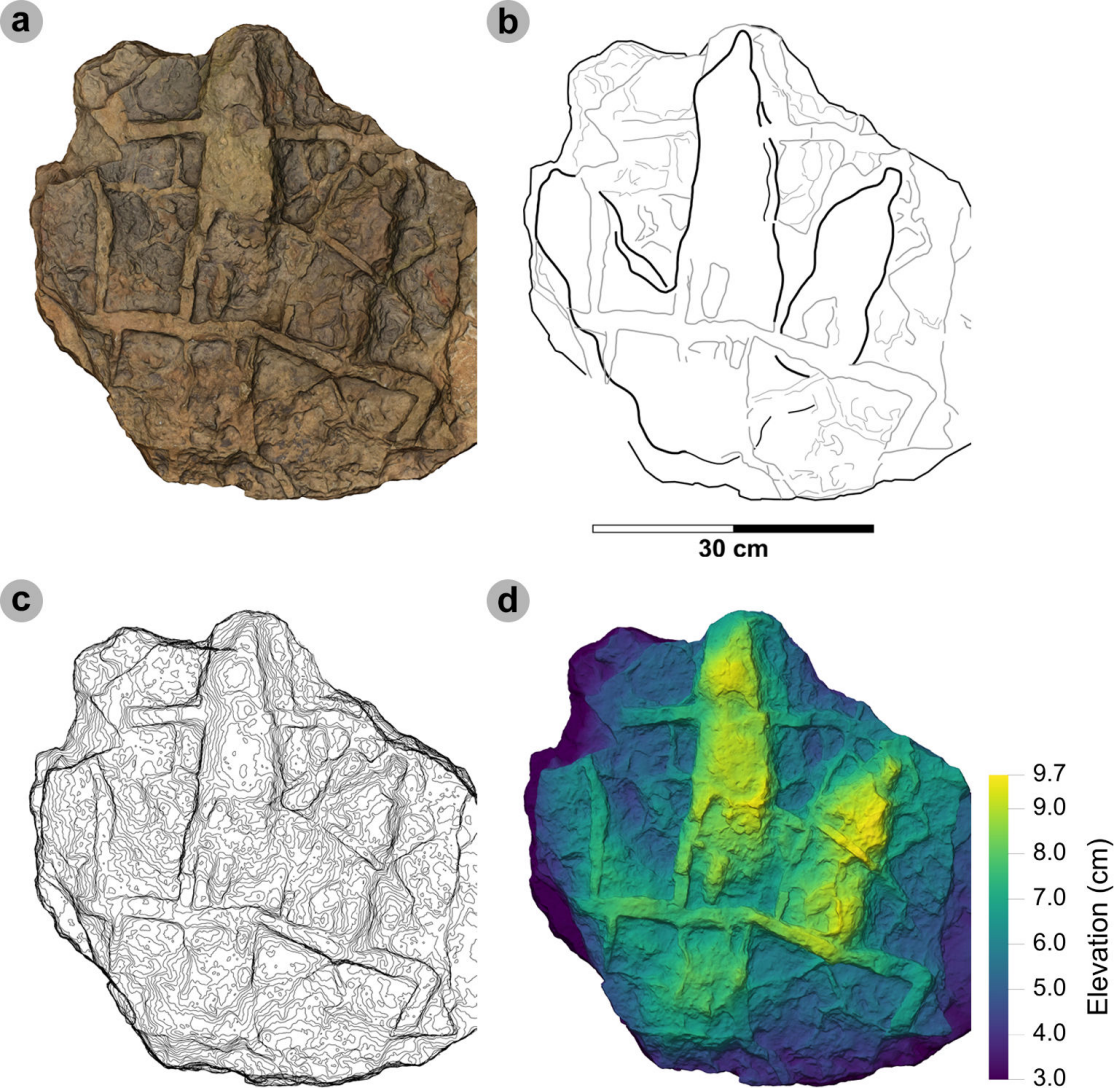
Digital representations of morphotype HBR_B1.1. (a) Textured orthophoto, (b) outline, (c) 2 mm contour map and (d) DEM of SM.1976.2002.004—the only presently known HBR_B1.1 track from Valtos. Desiccation cracks formed after the track was impressed during subaerial exposure.

**Material**: SM.1976.2002.004 (figure 5; table 2)

**Table 2.** Morphotype HBR_B1.1 (SM.1976.2002.004) track measurements.

						digit length (DL)			DL ratios			divarication angles				
specimen	PG	L/R	L	W	L/W	II	III	IV	III/II	III/IV	III/L	II–III	III–IV	II–IV	te	M
2002.004	1.5	R*	43.88	33.37	1.31	23.68	27.56	30.38	1.16	0.91	62.81	29.03	23.46	52.49	13.81	0.41

All lengths were measured in cm. The III/L ratio is expressed as a percentage. Divarication angles were measured in degrees. All track measurements were rounded to two decimal places.

**Referred material:** SM.1976.2010.001 (electronic supplementary material, figure S1, table S1)

*Note*: Morphotype diagnosis is based on ‘Morphotype 1 a’ from [22]. Morphotype description is based on SM.1976.2002.004—the only track in the current *ex situ* sampling from Valtos and Lúb Score diagnosed under HBR_B1.1.

#### 3.2.1. Diagnosis

Large-sized tridactyl footprint with weak to moderate l/w ratios (1.22−1.66) and mesaxony (0.36−0.52). Digits are broad, parallel-sided and distally taper toward elongated, subtriangular ungual marks. Track lengths typically range between 40 and 55 cm. Phalangeal pad margins are often discernible from digit margins and features a ‘X : 2 : 3 : 4’ formula. Digit iv features a large, sub-circular metatarsophalangeal pad. Digit ii–iv divarication angles are generally between 40° and 60°. Digit i is absent.

#### 3.2.2. Description

Large, asymmetric tridactyl footprint, 43.9 cm long by 33.4 cm wide. The l/w ratio (1.31) and mesaxony (0.41) fall within the lower ranges of HBR_B1. Digits are broad and parallel-sided with a faint ‘X : 2 : 3 : 4’ phalangeal pad formula. Digit iii occupies 62.8% of the track length. The digit iii/ii ratio (1.16) is larger than its digit iii/iv equivalent (0.91). The overall digit ii–iv divarication angle is 52.5°.

#### 3.2.3. HBR_B1.1 material description

SM.1976.2002.004 is currently the only definitive example of morphotype HBR_B1.1 from the investigated track sites and has a preservation grade of 1.5 (figure 5). The positive relief track originates from the lower unit of the Valtos Sandstone Formation (bed 18) and is composed of a fine-grained, well-indurated sandstone. It falls into the lower range of HBR_B1 morphotype in terms of both l/w ratio and mesaxony. Although vague, a ‘X : 2 : 3 : 4’ phalangeal pad formula is distinguished from broad digit margins. Their definition gradually declines toward digit iv. The digits are parallel-sided across much of their length, particularly digit iii, and distally taper toward subtriangular-shaped, elongated ungual marks, the clearest of which are anteromedially oriented on digits ii–iii. A partial large tridactyl track from Lùb Score (SM.1976.2010.001), resembling HBR_B1.1, is discussed and figured in electronic supplementary material, figure S1.

### 3.3. HBR_B1.2

**Figure 6.**
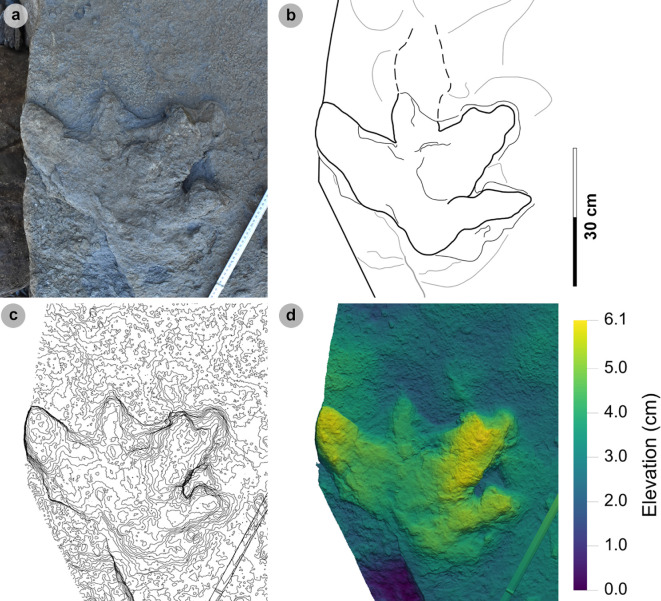
Photographic and digital representations of VA12. (a) Photograph, (b) outline, (c) 2 mm contour map and (d) DEM of VA12. Digit iii is most eroded distally, while digits ii and iv are complete and appear much broader than for other HBR_B1 morphotype subgroups. A distinctive anterolaterally oriented hallux possesses two phalangeal pads and an ungual mark.

**Material**: VA12 (figure 6; table 3)

**Table 3.** Limited track measurements of VA12.

						digit length (DL)			DL ratios			divarication angles				
specimen	PG	L/R	L	W	L/W	II	III	IV	III/II	III/IV	III/L	II–III	III–IV	II–IV	te	M
VA12	1	R*	n/a	39.40	n/a	27.60	n/a	27.60	n/a	n/a	n/a	n/a	n/a	73.10	n/a	n/a

All lengths were measured in cm. Divarication angles were measured in degrees. All track measurements were rounded to two decimal places.

*Note*: Morphotype diagnosis and description are based on VA12—the only representative track of this morphotype. As a result, the full characteristics of missing morphologies, such as digit iii, are not currently known for this morphotype.

#### 3.3.1. Diagnosis

Grouped under HBR_B1 as track features broad, sub-parallel-sided digits. Differentiated from HBR_B1.1 with the presence of a tapering digit i and broad heel region. Digits are broad and lack metatarsophalangeal pads. Digit i features a double-padded hallux with a tapering ungual mark. Digits ii and iv are equal in length.

#### 3.3.2. Description

Deeply impressed, partial positive relief tetradactyl footprint missing most of digit iii. Track is approximately 39.4 cm wide with very broad digits. Digit margins are poorly defined but show signs of tapering. Digits ii and iv are equal in length (27.6 cm). Digit i is pronounced and anterolaterally directed with two longer than wide, oval-shaped phalangeal pads and a broad, triangular-shaped ungual mark. Divarication angles between digits i–ii are 32.1° and digits ii–iv are 73.1°.

#### 3.3.3. HBR_B1.2 material description

VA12 is presently the only recorded HBR_B1.2 track. The track occurs within a well indurated, finely laminated, fine-grained sandstone correlated to bed 16 of the lower Valtos Sandstone Formation. The morphotype is uniquely characterized by broader, tapering digits and possesses a hallux with two, longer than wide, oval-shaped phalangeal pads (figure 6). Similar experimental tracks with broad digit margins are reported from substrates with low competency [66]. At the very least, these morphological characteristics may indicate that the track-bearing surface was competent enough to permit the impression of a hallux and metatarsal region—similar to a large theropod track reported by [67]. VA12 thus contrasts most other assessed tracks, which generally lack a hallux.

### 3.4. HBR_B1.3

**Figure 7.**
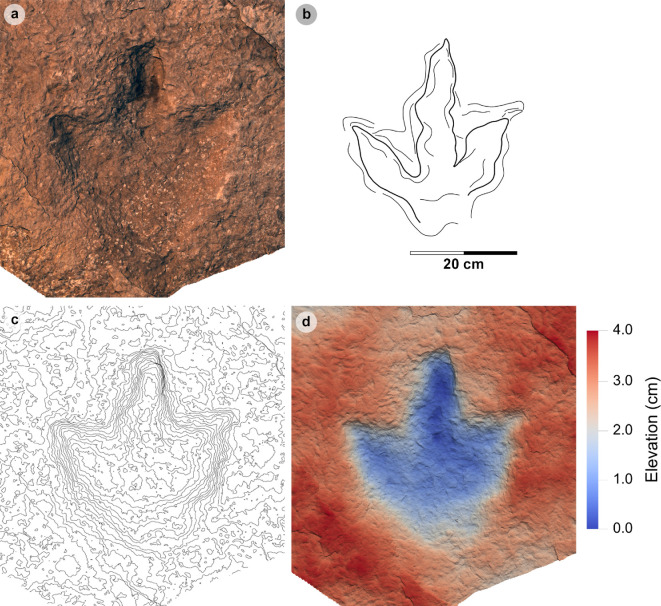
Digital representations of SM.1976.2002.008a. (a) Photograph, (b) outline, (c) 2 mm contour map and (d) DEM. SM.1976.2002.008a represents the only example of the HBR_B1.3 morphotype subgroup from the investigated localities at present. The track is distinguished by an approximately 30 cm track length, sub-parallel-sided digit margins and poorly defined digit iv metatarsophalangeal pad. Like HBR_B1.1, digit iii occupies approximately 60% of the total track length and the l/w ratio is weak (approx. 1.30).

**Material**: SM.1976.2002.008a (figure 7; table 4)

**Table 4.** Measurements for HBR_B1.3.

						digit length (DL)			DL ratios			divarication angles				
specimen	PG	L/R	L	W	L/W	II	III	IV	III/II	III/IV	III/L	II–III	III–IV	II–IV	te	M
2002.008 a	1	L	31.78	24.67	1.29	15.85	19.11	17.22	1.21	1.11	60.13	33.50	38.55	72.05	13.62	0.55

All lengths were measured in cm. The III/L ratio is expressed as a percentage. Divarication angles were measured in degrees. All track measurements were rounded to two decimal places.

*Note:* Morphotype diagnosis expands on ‘Morphotype 1 c’ from [22]. Morphotype description is based on SM.1976.2002.008a.

#### 3.4.1. Diagnosis

Characterized by low l/w ratios (1.26−1.32), moderate mesaxony (0.51−0.55) and sub-parallel-sided broad digits which taper toward elongated, triangular ungual marks. Differentiated from morphotypes HBR_B1.1−1.2 with an approximately 30 cm track length and moderately slender digits. The digit iv metatarsophalangeal pad is not well defined. Digit ii–iv divarication angles are >60°.

#### 3.4.2. Description

A large, asymmetric tridactyl footprint, 31.8 cm in length and 24.7 cm wide with a 1.29 l/w ratio. The track is moderately mesaxonic (0.55) with a wide digit ii–iv divarication angle of 72.1°. Digit iii is the longest digit, followed by digits iv and ii. Digits are moderately slender, sub-parallel-sided and distally taper toward elongated, triangular-shaped ungual marks. Digit iii occupies 60.1% of the total track length.

#### 3.4.3. HBR_B1.3 material description

SM.1976.2002.008a is a negative relief track and the only presently known example of morphotype HBR_B1.3 from the investigated localities (figure 7). The track occurs in a horizon of upper Valtos Sandstone Formation composed of a fine-grained shaley sandstone packed with disarticulated *Neomiodon* bivalves. In cross-section, the slab is finely laminated, with increased induration and fewer *Neomiodon* bivalves in older horizons. The lowermost horizon preserves desiccation cracks and contains positive relief HBR_B2.1 tracks (SM.1976.2002.008b). Like SM.1976.2002.004 (morphotype HBR_B1.1), the l/w ratios of SM.1976.2002.008a are approximately 1.30—the lower range of HBR_B1, and digit iii occupies approximately 60% of the total track length. The track is distinguished under a separate subgroup due to differences in digit ii–iv divarication angle (narrower in HBR_B1.1 at 52.5°), moderately slender digits with sub-parallel-sided margins (broader and parallel-sided in HBR_B1.1), and a poorly defined digit iv metatarsophalangeal pad. The digit margins are rounded and smooth, gradually flattening out toward the substrate surface and lack phalangeal pads. Unlike other HBR_B1 tracks, the lateral digit ungual marks are subtly anterolaterally oriented.

### 3.5. HBR_B1.4

**Figure 8.**
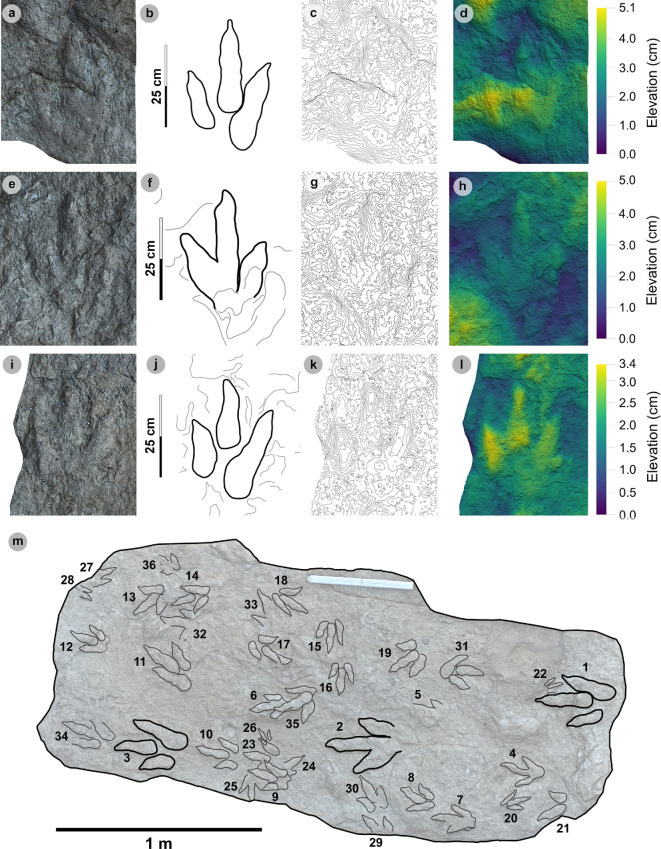
Digital representations of VA10-1−3. Textured orthophotos, outlines, 2 mm contour maps and DEMs are respectively represented for: (a*–*d) VA10-1, (e*–*h) VA10-2, (i–l) VA10-3. Although morphologies such as phalangeal pads and ungual marks are distinguished on most digits, the track margins appear worn. (m) Outline map overlaid onto textured orthomosaic of VA10 surface shows HBR_B1.4 tracks (bold) in context with one another and surrounding HBR_B2 and HBR_B3 tracks.

**Material**: VA10-1–3 (figure 8; tables 5–7)

**Table 5.** Measurements for VA10-1−3.

						digit length (DL)			DL ratios			divarication angles				
specimen	PG	L/R	L	W	L/W	II	III	IV	III/II	III/IV	III/L	II–III	III–IV	II–IV	te	M
VA10-1	1	L*	35.75	20.37	1.76	15.44	24.59	22.45	1.59	1.10	68.78	14.21	14.79	29.00	14.49	0.71
VA10-2	1	R*	33.95	21.70	1.56	18.95	23.25	21.18	1.23	1.10	68.48	20.53	23.98	44.51	13.52	0.62
VA10-3	1.5	L*	32.79	20.81	1.58	15.08	17.93	25.47	1.19	0.70	54.68	16.28	19.24	35.52	10.36	0.50
***average***	***1.17***	**—**	***34.16***	***20.96***	***1.63***	***16.49***	***21.92***	***23.03***	***1.34***	***0.97***	***63.98***	***17.01***	***19.34***	***36.34***	***12.79***	***0.61***

All lengths were measured in cm. The III/L ratio is expressed as a percentage. Divarication angles were measured in degrees. All track measurements were rounded to two decimal places.

**Table 6.** Trackway measurements for VA10-1−3.

specimen	L	h	P	λ	WAP	α	γ
VA10-1	35.75	1.43	1.05	2.1	10.2	5.56	n/a
VA10-2	33.95	1.36	1.06	n/a	n/a	5.52	168.91
VA10-3	32.79	1.31	n/a	n/a	n/a	n/a	n/a
***average***	***34.16***	***1.37***	***1.06***	***n/a***	***n/a***	***5.54***	***n/a***

All values were rounded to two decimal places. Total track length (L) and width of pes angulation (WAP) were measured in cm. Hip height (h), pace (P) and stride (λ) lengths were measured in m. Angles of rotation (α) and pace angulation (γ) were measured in degrees.

**Table 7.** Velocity and gait measurements for VA10-1−3.

track λ	L/R	L	h	λ	λ/h	V (A)	V (RT)
VA10-1−3	L	34.16	1.37	2.10	1.54	1.88	1.70

All values were rounded to two decimal places. Track length (L) represents the trackway average and was measured in cm. Hip height (h) and stride length (λ) were measured in m. Velocity was measured in m s^−1^. ‘V (A)’ is measured according to [62] and ‘V (RT)’ according to [63].

#### 3.5.1. Diagnosis

Classified under HBR_B1 due to large track size, broad and parallel-sided digits, longer than wide oval-shaped phalangeal pads (the digit iv metatarsophalangeal pad is the largest pad), relatively parallel-sided digit margins and elongated ungual marks. Distinguished from other HBR_B1 subgroups with track lengths in the 30−40 cm range, elongated l/w ratios >1.50 and narrower digit ii–iv divarication angles <45°.

#### 3.5.2. Description

Large asymmetric tridactyl footprint, 32.8−35.8 cm long and 20.4−21.7 cm wide. Tracks possess moderate-pronounced l/w ratios (average 1.63) and mesaxony (average 0.61). Digit margins are relatively parallel sided with non-discretely separated phalangeal pads and an unclear formula (X : 2 : 3? : ?). The digit iv metatarsophalangeal pad is the largest pad, longer than wide and oval shaped. Digits ii–iv have divarication angles between 29.0° and 44.5°.

#### 3.5.3. HBR_B1.4 material description

HBR_B1.4 is known from a single trackway of three positive relief tracks (VA10-1−3) across a horizon of track-rich, medium- to coarse-grained sandstone, forming part of bed 25 of the lower Valtos Sandstone Formation (figure 8). Although worn, the parallel-sided digit margins highlight phalangeal pads (with an unknown complete formula) and show the digits distally tapering toward sharp, elongated ungual marks—similar to other HBR_B1 subgroups. HBR_B1.4 is metrically distinguished from other HBR_B1 subgroups due to moderate-pronounced l/w ratios (1.56−1.76) and mesaxony (0.50−0.71) and narrower digit ii–iv divarication angles (29.0°−44.5°).

VA10-1−3 form a clear 2.1 m long stride with a consistent pace of 1.05−1.06 m, narrow WAP (10.2 cm) and angle of rotation (5.52°−5.56°) (table 6). Trackway measurements imply the HBR_B1.4 trackmaker travelled at a walking gait (1.54) with an estimated velocity of 1.70−1.88 m s^−1^ (6.12−6.77 km h^−1^ = 3.80–4.21 mph) (table 7). The trackmaker likely stood at approximately 1.37 m to the hip.

#### 3.5.4. HBR_B1 ichnotaxonomy

The >40 cm track length, relatively low l/w ratio, moderate mesaxony and broad digits of HBR_B1.1 most resemble the ichnogenus *Megalosauripus. Megalosauripus uzbekistanicus* tracks possess similarly weak l/w ratios (~1.20), mesaxonic indices (0.40) and digit ii–iv divarication angles <70° [68]. A study by [69] described the digit margins of *Megalosauripus* as ‘parallel-sided’—a character also observed in HBR_B1.1. The *Megalosauripus-*referred morphotype ‘Bxviii’ from the Cleveland basin also featured similar low l/w ratios (1.24−1.34) and moderate digit ii–iv divarication angles (45°−70°) [67,70].

Morphotype HBR_B1.2 contrasts with broader digits but suffers from erosion and other taphonomic processes and is not referable to an ichnotaxon. Despite this, HBR_B1.2 features a distinctive laterally oriented padded hallux and pronounced heel. The hallux of HBR_B1.2 differs from the equivalent ungual only impression on some *Megalosauripus uzbekistanicus* tracks by additionally featuring two oval-shaped phalangeal pads. The pads and subtly rounded metatarsal region of HBR_B1.2 are reminiscent of those of *Boutakioutichnium atlasicus* [71]. Unlike *Boutakioutichnium*, however, the heel region of HBR_B1.2 rests below digit ii–iii rather than digit iv. A possible hallux is reported in a similar position on the deeply impressed track referred to *Megalosauripus* isp. [67].

The characteristics of morphotype HBR_B1.3 overlap with multiple ichnotaxa. Although the digit margins of HBR_B1.3 are sub-parallel-sided, they can also be construed as subtly spindle-shaped like *Eubrontes*—a character noted in Early Jurassic North American tracks by [59]. However, the weak l/w ratio (1.29), moderate mesaxony (0.55) and approximately 60% digit iii/l ratio of HBR_B1.3 more closely occupy the ranges of *Megalosauripus* and HBR_B1.1. Unlike typical large-sized *Megalosauripus* tracks, and HBR_B1.1, HBR_B1.3 possesses moderately slender digits and is <40 cm in length (31.8 cm). Similar tracks reported from Portugal and Morrocco are more narrowly divaricated between digits ii–iv (<60°) but possess similar moderately slender digit thicknesses [69,72]. These metrics, in combination with a wide 72.1° digit ii–iv divarication angle, also resemble those of *Kayentapus* [73,74]. Despite this, the digits of morphotype HBR_B1.3 are broader than *Kayentapus* tracks [74,75]. On balance, HBR_B1.3 is most consistent with *Megalosauripus* despite the observed differences in track length and divarication angle.

Morphotype HBR_B1.4 possesses broader digits with more ‘parallel-sided’ margins like HBR_B1.1, with a pronounced, oval-shaped digit iv metatarsophalangeal pad similar to *Megalosauripus* [69]. Despite this, HBR_B1.4 tracks are <40 cm in length and feature interdigital angles which fall below the 22°−40° range reported by [31]. Based on its track length, pronounced l/w ratios (>1.50), moderate digit iii/l ratio ranges (54.7–68.8%), narrower digit ii−iv divarication angles (29.0°−44.5°) and lack of discretely separated phalangeal pads, HBR_B1.4 is similar to *Therangospodus* [76]. *Therangospodus* is commonly found amongst *Megalosauripus* tracks [68,77]. Middle–Late Jurassic *Therangospodus* tracks from Utah and Uzbekistan exhibited large l/w ratio ranges, some pronounced (1.10−1.77), and shorter approximately 30 cm lengths similar to HBR_B1.4 [76]. At the Middle Jurassic aged Moab megatracksite in North America, Lockley *et al*. [76] suggest *Therangospodus* as an ichnospecies is very similar in shape to *Megalosauripus*, and the two may have been made by a similar trackmaker.

Overall, HBR_B1.1 most resembles *Megalosauripus* isp. due to its approximately 40 cm length, weak l/w ratios (1.32), moderate mesaxony (0.41) and digit ii–iv divarication angle (52.5°). HBR_B1.2 is the most equivocal of the morphotype subgroups and is not strongly consistent with present ichnotaxa. HBR_B1.3 is, tentatively, most likely *Megalosauripus* due to its sub-parallel-sided digits, similar weak l/w ratios (1.29) and moderate mesaxony (0.55) despite possessing slenderer digits and a wider digit ii–iv divarication angle. HBR_B1.4 is most similar to *Therangospodus* based on <40 cm lengths, pronounced l/w ratios (1.63) and mesaxony (0.61) and <45° digit ii–iv divarication angle.

### 3.6. HBR_B2.1

**Figure 9.**
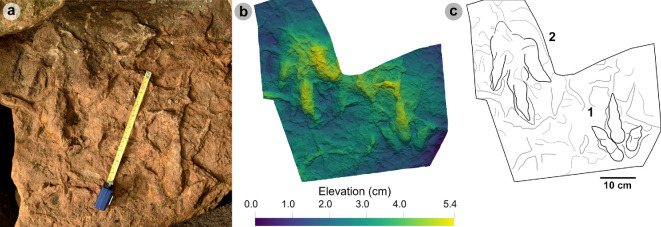
Photographic and digital representations of VA11. (a) Photograph, (b) DEM and (c) labelled outline diagram. The tracks illustrate the variation in metrics (e.g. l/w ratios, divarication angle) and morphology (e.g. digit thickness) within HBR_B2.1. VA11-1 (right) represents a ‘slender’ form, with narrowly thick tapering digits, elongated ungual marks and low-moderate l/w ratios <1.50. VA11-2 (left) represents a ‘broad’ form, characterized by digits with a more pronounced spindle-shape and subtly broader thickness (usually by 1 cm). Tracks possess more pronounced l/w ratios >1.50 and generally narrower digit ii–iv divarication angles <50°.

**Figure 10.**
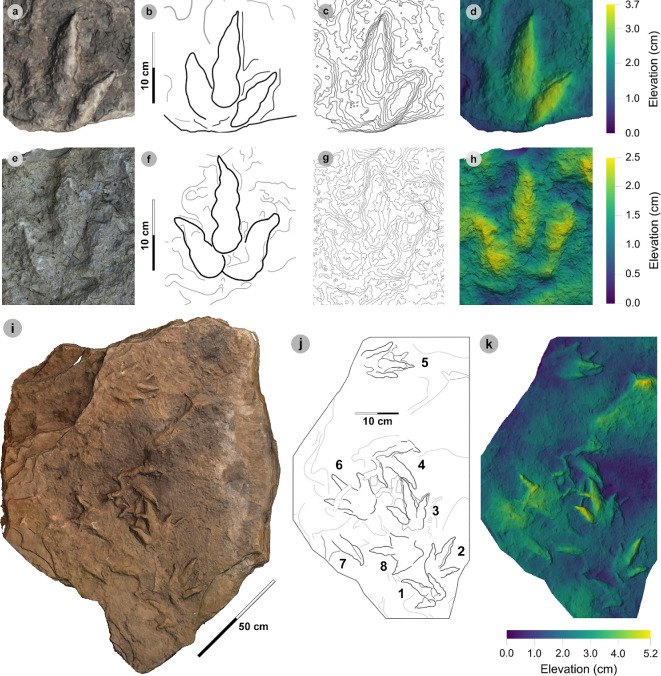
Digital representations of ‘slender’ form HBR_B2.1 tracks from Valtos. Textured orthophotos, outlines, 2 mm contour maps and DEMs are accordingly represented for: (a*–*d) SM.1976.2016.001b-1, (e–h) VA09-1 and (i–k) SM.1976.2002.008b. Note that SM.1976.2016.001b-1 and VA09-1 are positive relief representations of left tracks. The tracks represent a form of HBR_B2.1 characterized by slender digits, l/w ratios <1.50 and iii/L ratios >70%. The track-bearing surface of SM.1976.2002.008b contains at least eight positive relief tracks (labelled on outline diagram) bearing multiple directions. Tracks 1−3 were used to characterize HBR_B2.1 due to their sharp digit margins with distinguishable phalangeal pads and sharp ungual marks.

**Figure 11.**
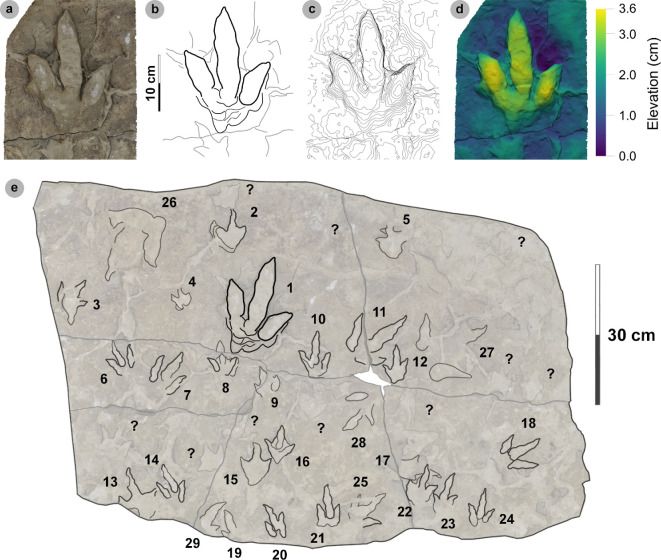
Photographic and digital representations of SM 1976.2002.007-1. (a) Photograph, (b) outline, (c) 2 mm contour map and (d) DEM of SM 1976.2002.007-1 isolated from main slab. (e) SM 1976.2002.007-1 (bold) in context to smaller HBR_B3 tracks. Note that all tracks face a similar direction.

**Figure 12.**
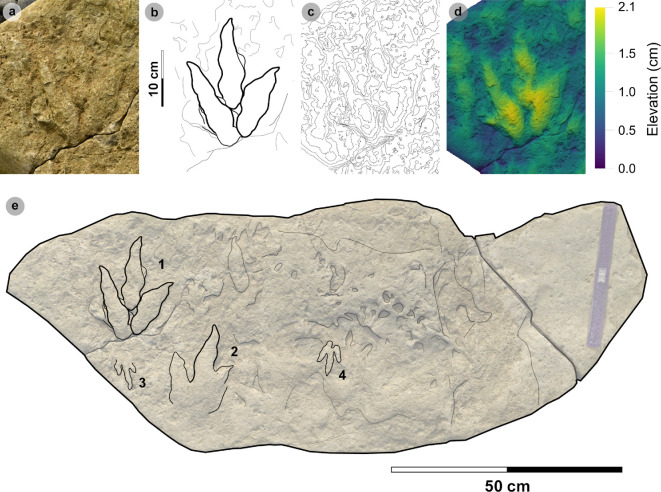
Photographic and digital representations SB02-1. (a) Photograph, (b) outline, (c) 2 mm contour map and (d) DEM of SB02-1 isolated from main slab. (e) SB02-1 in context to other tracks represented on outlined textured orthomosaic of full track-bearing surface. Note that both HBR_B3 tracks face opposite directions to SB02-1−2.

**Figure 13.**
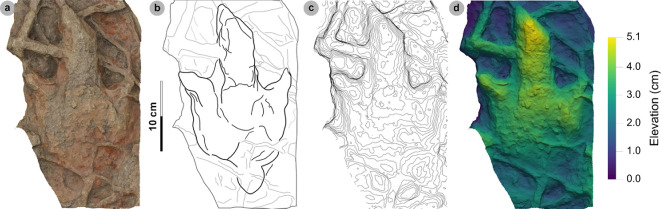
Digital representation of SM.1976.2002.003. (a) Textured orthophoto, (b) outline, (c) 2 mm contour map and (d) DEM. Faint phalangeal pad margins can be seen on all digits.

**Figure 14.**
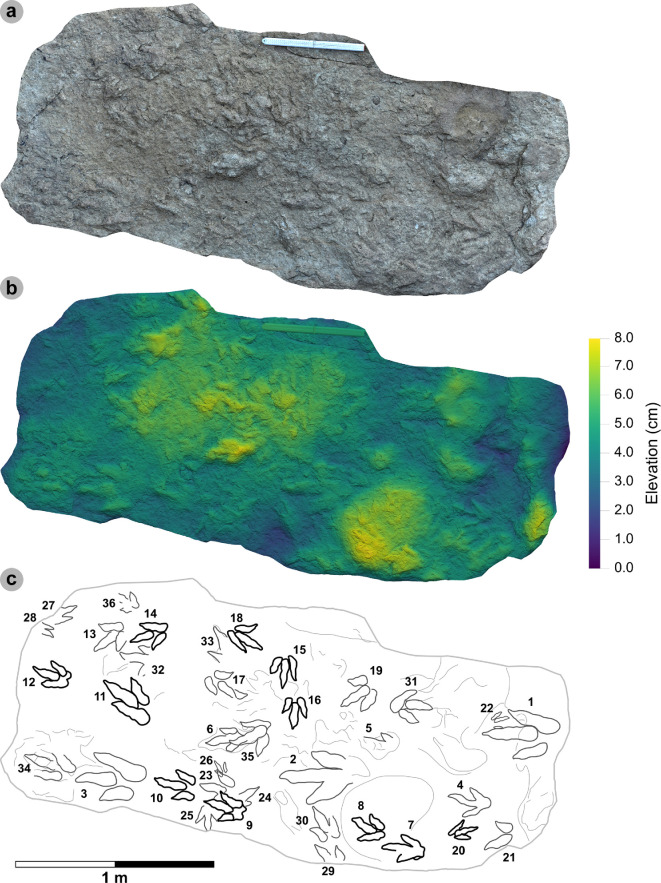
Digital representations of HBR_B2.1 tracks on VA10. (a) Textured orthophoto of entire track-bearing surface with 50 cm scale bar, (b) DEM and (c) outline with numbered tracks. Tracks characterized as HBR_B2.1 are bold on the outline diagram. The positive relief tracks bear multiple directions, some form track associations and exhibit short paces or strides.

**Material**: SM.1976.2002.003, SM.1976.2002.007-1, SM.1976.2002.008b-1–3, SM.1976.2011.001-1, SM.1976.2016.001b-1, VA09-1, VA10-7–12, 14–16, 18, 20, VA11-1–2 and SB02-1 (figures 9–14; table 8)

**Table 8.** Track measurements used to characterize HBR_B2.1.

						digit length (DL)			DL ratios			divarication angles				
specimen	PG	L/R	L	W	L/W	II	III	IV	III/II	III/IV	III/L	II–III	III–IV	II–IV	te	M
2002.003	1	L*	22.59	13.39	1.69	11.97	15.00	15.42	1.25	0.97	66.40	22.22	23.01	45.23	7.86	0.59
2002.007-1	2	R*	21.00	12.21	1.72	11.25	14.15	13.95	1.26	1.01	67.38	33.11	18.95	52.06	7.89	0.65
2002.008b-1	1.5	L*	17.07	12.57	1.36	6.37	12.42	11.46	1.95	1.08	72.76	31.77	21.90	53.67	7.73	0.61
2002.008b-2	1.5	R*	21.43	13.80	1.55	8.19	16.70	11.28	2.04	1.48	77.93	38.38	18.98	57.36	9.14	0.66
2002.008b-3	2	L*	22.45	14.73	1.52	9.00	15.76	13.85	1.75	1.14	70.20	28.77	24.29	53.06	10.63	0.72
2011.001-1	1.5	L	16.07	11.13	1.44	7.48	11.46	11.78	1.53	0.97	71.31	23.24	21.50	44.74	5.21	0.47
2016.001b-1	2	L*	14.82	10.71	1.38	6.68	11.60	6.62	1.74	1.75	78.27	23.24	26.20	49.44	7.07	0.66
VA09-1	1.5	L*	15.34	11.37	1.35	8.11	11.74	9.15	1.45	1.28	76.53	34.68	23.63	58.31	7.59	0.67
VA10-7	1.5	R*	20.33	11.47	1.77	12.48	13.79	12.62	1.10	1.09	67.83	28.61	26.69	55.30	8.17	0.71
VA10-8	1.5	R*	16.88	12.08	1.40	8.83	12.14	12.86	1.37	0.94	71.92	13.46	39.25	52.71	6.25	0.52
VA10-9	1.5	L*	18.98	10.87	1.75	11.04	12.83	12.80	1.16	1.00	67.60	22.19	13.19	35.38	7.45	0.69
VA10-10	1.5	R*	20.19	11.71	1.72	9.36	15.26	9.67	1.63	1.58	75.58	14.96	15.33	30.29	10.24	0.87
VA10-11	1.5	R*	27.46	13.66	2.01	11.92	18.60	17.92	1.56	1.04	67.73	21.66	12.44	34.10	10.13	0.74
VA10-12	1.5	R*	16.54	11.20	1.48	10.97	11.09	13.72	1.01	0.81	67.05	25.64	18.02	43.66	5.42	0.48
VA10-14	1.5	L*	18.56	11.56	1.61	8.65	10.51	11.88	1.22	0.88	56.63	30.60	26.30	56.90	7.75	0.67
VA10-15	1.5	R*	16.92	11.13	1.52	8.51	13.60	11.02	1.60	1.23	80.38	21.05	10.98	32.03	6.13	0.55
VA10-16	1.5	R*	14.58	10.71	1.36	6.34	13.13	9.40	2.07	1.40	90.05	20.02	23.89	43.91	6.95	0.65
VA10-18	1.5	L*	16.84	10.88	1.55	8.61	13.14	11.84	1.53	1.11	78.03	29.11	19.69	48.80	6.08	0.56
VA10-20	1.5	R*	13.12	7.92	1.66	6.77	10.05	8.62	1.48	1.17	76.60	19.85	25.06	44.91	4.70	0.59
VA11-1	2	R*	15.96	11.85	1.35	7.87	11.70	10.17	1.49	1.15	73.31	32.59	28.06	60.65	7.96	0.67
VA11-2	1	L*	24.77	12.54	1.98	11.25	16.42	14.55	1.46	1.13	66.29	23.23	13.39	36.62	9.71	0.77
SB02 -1	2	R*	21.59	14.09	1.53	12.32	14.96	15.64	1.21	0.96	69.29	30.17	23.10	53.27	7.98	0.57
***average***	***1.57***	**—**	***18.80***	***11.89***	***1.58***	***9.27***	***13.46***	***12.10***	***1.49***	***1.14***	***72.23***	***25.84***	***21.54***	***47.38***	***7.64***	***0.64***

All lengths were measured in cm. The III/L ratio is expressed as a percentage. Divarication angles were measured in degrees. All track measurements were rounded to two decimal places.

**Referred material**: SM.1976.2000.001-2, SM 1976.2002.008b-4–8, SM 1976.2006.002a-b, SM 1976.2006.003-1, SM 1976.2006.008a-b, SM 1976.2006.013-1, SM 1976.2006.015, SM 1976.2006.016, SM 1976.2006.017b, SM 1976.2021.001b-1, SM 1976.2022.004, SM 1976.2022.008, SM.1976.2022.025, SM.1976.2023.001, SM.1976.2024.001-1, SM.1976.2024.001-2, VA08, VA09-3, VA10-4, 6, 17, 30, 31, 34, VA14-1–2, 5, SB022 (electronic supplementary material, table S2)

#### 3.6.1. Diagnosis

Classed under HBR_B2 as digits are moderately slender, spindle-shaped and distally taper toward narrow, elongated ungual marks. Track lengths typically range between approximately 15 and 25 cm (within a small–medium range). Tracks are moderately to strongly mesaxonic (indices often >0.50) with l/w ratios >1.30. Digits ii–iv are divaricated between approximately 30° and 60°.

#### 3.6.2. Description

A small- to medium-sized asymmetric tridactyl footprint of 13.1−27.5 cm in length and 7.9−14.7 cm wide (l/w ratios 1.35−2.01) with moderate-pronounced mesaxony (0.47−0.77). Digits are spindle-shaped, poorly separated and distally taper towards short or elongated gracile triangular ungual marks. On average, digit iii occupies 72.2% of the track length and is the longest digit. Digit iii/ii ratios are larger (average 1.49) than digit iii/iv ratios (average 1.14). Phalangeal pad margins are often weakly defined and vary considerably. Digits ii–iv are divaricated between 30.3° and 60.7° (average 47.4°).

#### 3.6.3. HBR_B2.1 material description

Morphotype HBR_B2.1 is frequently recorded throughout the lower and upper Valtos Sandstone Formation, while scarcer in the Kilmaluag Formation. When digit margins are sharply defined, some HBR_B2.1 tracks can be distinguished into ‘slender’ and ‘broad’ forms—as illustrated on VA11 (figure 9). The slender form, represented by VA11-1, is best known from the Valtos Sandstone Formation. The digits are spindle-shaped, narrow in thickness and shallowly taper across much of their length toward elongated ungual marks. Although the digits are poorly separated, digit ii appears more separated from the posterior margins of digit iii than digit iv. Metrically, this ‘slender form’ is distinguished by l/w ratios <1.50 and iii/L ratios generally >70%. Individual examples include SM.1976.2016.001b-1 and VA09-1 (figure 10a–h). The clearest track association of this form occurs on SM.1976.2002.008b (figure 10i–k).

The second ‘broad’ form, represented by VA11-2, is less common but recorded in both the Valtos Sandstone and Kilmaluag Formations. This form is similar to the ‘slender’ form with tapering spindle-shaped digits but differs with subtly broader digit thicknesses (and phalangeal pads), pronounced l/w ratios>1.50 and generally narrower digit ii–iv divarication angles <50°. Some of the best examples from Lùb Score occur on track-rich surfaces. SM 1976.2002.007-1 is, uniquely, surrounded by several examples of smaller HBR_B3 tracks which bear a similar direction to it (figure 11). SB02-1 occurs opposite a similarly sized referred HBR_B2.1 track (SB02-2) and near two smaller HBR_B3 tracks facing the opposite direction (figure 12). Examples from Valtos feature more pronounced, elongated ungual marks compared to the shorter triangular equivalents of those from Lùb Score, e.g. SM.1976.2002.003 (figure 13).

Despite their distinctions, both HBR_B2.1 variants are considered under one subgroup due to overlapping length ranges, digit iii/l ratios (average 72.2%), mesaxony (average 0.64), divarication angles (average 47.4°) and tapering, spindle-shaped digits. The clearest demonstration of morphotype variation is observed on the medium- to coarse-grained, sandy limestone (bed 25) surface of VA10—which contains 11 diagnostic, positive relief HBR_B2.1 tracks (a further six are referred) with worn track margins (figure 14). HBR_B2.1 dominates the surface over all other present morphotype subgroups (including HBR_B1.4 and HBR_B3.1−3.2) with tracks bearing multiple directions (see §4.3 for interpretation). Due to worn track margins, it is generally harder to confidently distinguish between the two subtle HBR_B2.1 variants on VA10. Despite this, most have suggestive features of the second variant, i.e. l/w ratios >1.50, digit iii/L ratios <70% and digit ii–iv divarication angles <50°. Regardless, VA10 provides insight into the movements deployed by trackmakers capable of registering HBR_B2.1 tracks. Associated tracks exhibit short paces and strides: VA10-7−8 (both right tracks) strode 21.6 cm and VA10-9−10 paced 25.5 cm. VA10-15−16 (also right tracks) strode 20.1 cm. A possible trackway (VA10-4−6) comprises tracks which resemble HBR_B2.1 (notably VA10-4). Due to generally lacking clear digit margins (particularly track 5), the tracks were not used to characterize the subgroup—tracks 4 and 6 are referred to.

### 3.7. HBR_B2.2

**Figure 15.**
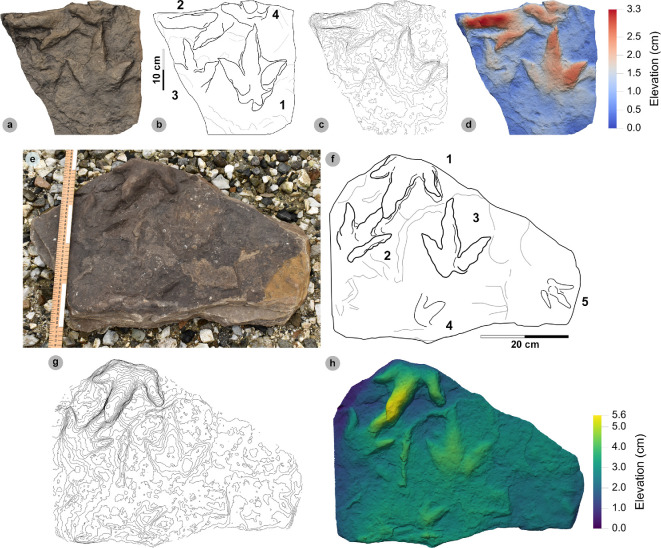
Photographic and digital representations of SM.1976.2000.001 and SM.1976.2006.005. Textured orthophotos or photograph with scale bar, labelled outlines, 2 mm contour maps and DEMs of tracks used to characterize HBR_B2.2: (a–d) track 1 in SM.1976.2000.001 and (e–h) track 3 in SM.1976.2006.005. Note tracks 1 and 2 share similar spindle-shaped, tapering, moderately slender digits to HBR_B2.1, but are referred to HBR_B2.2 due to their wide digit ii–iv divarication angle.

**Figure 16.**
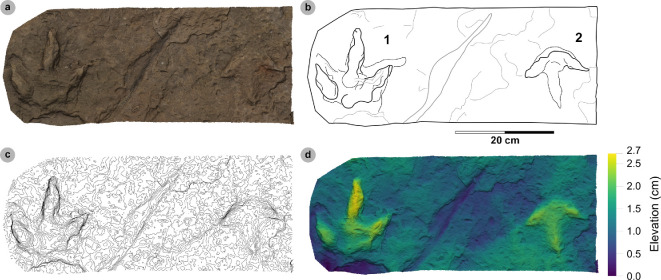
Digital representations of SM.1976.2006.006. (a) Textured orthomosaic, (b) labelled outline, (c) 1 mm contour map and (d) DEM of SM.1976.2006.006. The tracks are among those with the widest digit ii–iv divarication angles recorded from the Valtos Sandstone Formation and represent two different morphotypes. Track 1 was used to characterize HBR_B2.2, while track 2 is referred to HBR_B3.2 due to its smaller track length and slenderer digits.

**Figure 17.**
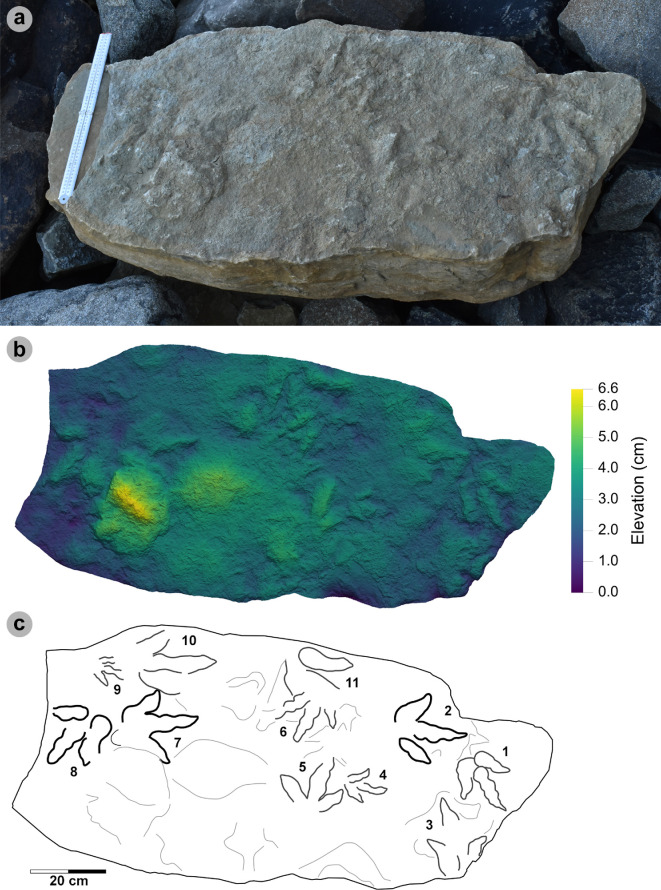
Photographic and digital representations of VA09. (a) Photograph with 50 cm scale bar, (b) DEM and (c) outline of VA09. HBR_B2.2 tracks are bold on outline diagram. Although worn in appearance, in part due to the medium-grained substrate composition, phalangeal pads are visible on the digit margins of VA09-2 and 7. The two tracks form a track association due to their relatively similar track and digit lengths, bearing and left–right alternation. Referred HBR_B3.1 tracks VA09-4 and 9 occur among these tracks and may also form a track association for similar reasons.

**Material**: SM.1976.2000.001-1, SM.1976.2006.005-3, SM.1976.2006.006-1, SM.1976.2006.007, SM.1976.2006.011-1, SM.1976.2016.001a-1, VA09-2, 7, 8, VA10-19 (figures 15–17, table 9)

**Table 9.** Measurements of HBR_B2.2 tracks.

						digit length (DL)			DL ratios			divarication angles				
specimen	PG	L/R	L	W	L/W	II	III	IV	III/II	III/IV	III/L	II–III	III–IV	II–IV	te	M
2000.001-1	2	R*	14.91	12.70	1.17	8.02	11.04	8.81	1.38	1.25	74.04	39.52	34.79	74.31	7.30	0.57
2006.005-3	1.5	L*	13.79	12.32	1.12	6.98	10.09	8.31	1.45	1.21	73.17	28.68	45.21	73.89	5.95	0.48
2006.006-1	1.5	L*	14.41	13.84	1.04	6.30	10.21	10.82	1.62	0.94	70.85	40.24	39.85	80.09	7.09	0.51
2006.007	1.5	L*	13.22	10.40	1.27	6.17	9.75	8.96	1.58	1.09	73.75	28.93	32.63	61.56	5.79	0.56
2006.011-1	2	L*	15.27	12.71	1.20	7.06	10.31	11.20	1.46	0.92	67.52	47.52	26.86	74.38	6.08	0.48
2016.001a-1	1	R	17.60	13.55	1.30	7.43	12.54	8.13	1.69	1.54	71.25	39.86	37.13	76.99	7.70	0.57
VA09-2	1.5	R*	16.61	15.15	1.10	7.90	12.82	9.94	1.62	1.29	77.18	32.73	39.46	72.19	8.40	0.55
VA09-7	1.5	L*	19.93	16.00	1.25	11.45	13.46	13.80	1.18	0.98	67.54	33.13	31.45	64.58	7.60	0.48
VA09-8	1.5	R*	18.66	15.41	1.21	8.44	13.47	12.84	1.60	1.05	72.19	52.02	19.37	71.39	9.03	0.59
VA10-19	1.5	R*	17.88	14.76	1.21	8.19	15.46	11.78	1.89	1.31	86.47	23.54	20.67	44.21	7.86	0.53
***average***	***1.5***	**—**	***16.23***	***13.68***	***1.19***	***7.79***	***11.92***	***10.46***	***1.55***	***1.16***	***73.40***	***36.62***	***32.74***	***69.36***	***7.28***	***0.53***

All lengths were measured in cm. The III/L ratio is expressed as a percentage. Divarication angles were measured in degrees. All track measurements were rounded to two decimal places.

**Referred material:** SM 1976.2000.001-3, SM 1976.2002.001-2, SM 1976.2004.002b-1, SM 1976.2006.005-1-2, SM 1976.2006.014-1, SM 1976.2012.001-1, SM 1976.2021.001a-1, SM 1976.2021.002, SM 1976.2022.018a, VA09-5, VA10-13, 21, 23, 35, VA14-4 (electronic supplementary material, figure S2, table S3)

#### 3.7.1. Diagnosis

Tracks grouped under HBR_B2 due to their small–medium track length range and distally tapering spindle-shaped digits. Differentiated from HBR_B2.1 with subequal-low l/w ratios ≤1.30 and more strictly moderate mesaxonic indices (approx. 0.50). Digit ii–iv divarication angles are often >60°.

#### 3.7.2. Description

A small asymmetric, tridactyl footprint of 13.2−19.9 cm in length and 10.4−16.0 cm wide. Tracks are moderately mesaxonic (0.48−0.59) with weak l/w ratios ≤1.30 (1.04−1.30). Digits are spindle-shaped and taper toward short, narrow, subtriangular-shaped ungual marks. Digit iii is on average 73.4% of the track length and often the longest digit. Digit iii/ii ratios are larger (average 1.55) than digit iii/iv equivalents (average 1.16). Phalangeal pad margins are usually vaguely defined, although when visible, are arranged in ‘2 : 3 : 3’ or ‘2 : 3 : 2’ formulas. Digits ii–iv are often widely divaricated >60° (average 69.4°).

#### 3.7.3. HBR_B2.2 material description

HBR_B2.2 is most abundant in the Valtos Sandstone Formation. Equivalent tracks from the Kilmaluag Formation are scarce and all referred (see electronic supplementary material, appendix S1). Morphotype HBR_B2.2 differs from HBR_B2.1 with wider divarication angles (>60°), weaker l/w ratios (<1.30) and stricter moderate mesaxony (average 0.53). Despite these distinctions, the subgroups share similar track length ranges, digit iii/l ratios and tapering, spindle-shaped digits (e.g. VA09-1 and 2, see below). The mesaxonic range of HBR_B2.1 is broader but does overlap with HBR_B2.2.

Tracks with the sharpest track margins typically occur in horizons of medium-grained sandstone from an undetermined bed of the upper Valtos Sandstone Formation (e.g. SM.1976.2000.001-1 and SM 1976.2006.005-1−3) (figure 15). Unlike most HBR_B2 tracks, the lateral digit ungual marks of tracks 1−2 on SM 1976.2006.005 are sharply anterolaterally oriented but are similarly elongated and pronounced to most HBR_B2.1 tracks from Valtos. SM.1976.2006.006-1 is the most widely divaricated HBR_B2.2 track at 80.1° and faces the opposite direction to a smaller HBR_B3.2 track (SM.1976.2006.006-2) which has the largest recorded digit ii–iv angle recorded in this study at 107.8° (figure 16). Track 1 features a faint ‘2 : 3 : 3’ phalangeal pad formula based on digit margins. Unlike digit iv, digit ii appears subtly separated from digit iii. Similar digit separation is noted on diagnosable tracks present in the medium- to coarse-grained sandstones of bed 25 in specimen VA09 (tracks 2, 7, 8), which occur alongside an equivalent HBR_B2.1 track (VA09-1) (figure 17). Tracks 1 and 2 (HBR_B2.1 and 2.2, respectively) both feature slender digit thicknesses, and spindle-shaped, tapering digits. VA09-2 and 7 are associated due to their similar track and digit lengths, phalangeal pad formulas (visible for digits ii−iii) and bearing. Tracks 7 to 2 exhibit a left–right pace of 69.9 cm.

### 3.8. HBR_B2.3

**Figure 18.**
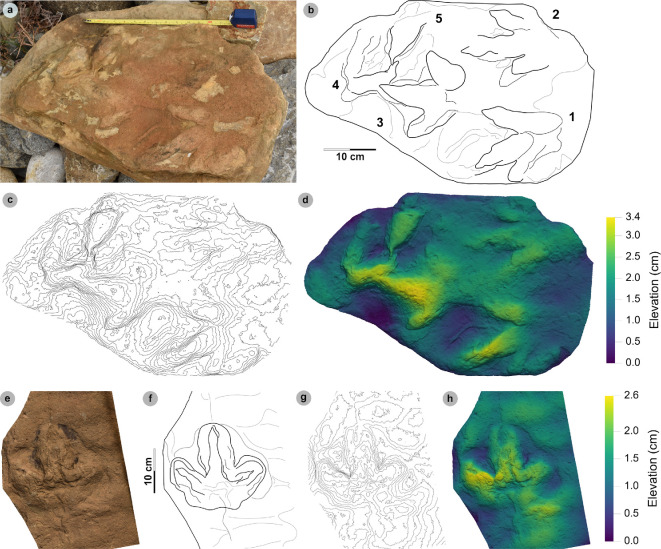
Photographic and digital representations of VA01 and VA06. (a) Photograph with 35 cm scale bar, (b) outline with track numbers, (c) 2 mm contour map and (d) DEM of VA01. Although the metrics of VA01-1−3 resemble those of other HBR_B2 subgroups, their enlarged digit margins and lack of clear ungual marks enabled their differentiation into HBR_B2.3. (e) Textured orthomosaic, (f) outline, (g) 2 mm contour map and (h) DEM of VA06. Both VA01 and VA06 are composed of medium-grained sandy limestones from bed 5. The tracks possess broad and round outer digit margins, with faint partial inner margins which resemble penetration track margins.

**Figure 19.**
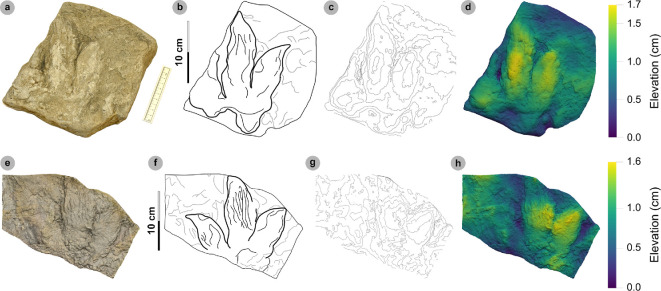
Photographic and digital representations of HBR_B2.3 tracks from Lùb Score. (a) Photograph with 10 cm scale bar, (b) outline, (c) 2 mm contour map and (d) DEM of GLAHM 114831. The track was figured in [65] under the Cleveland basin morphotype Bv. Digit iii features several partial inner sinuous digit margins within broad digits. (e) Textured orthomosaic, (f) outline, (g) 2 mm contour map and (h) DEM of SM.1976.2022.019. Although partial, the track characterizes HBR_B2.3 with round and broad digits with several inner sinuous margins within—most noticeable on a partial digit iii. Vague phalangeal pads may be present as bulges on the digit margins of both tracks.

**Material:** GLAHM 114831, GLAHM 152380, SM.1976.2002.001-1, SM 1976.2021.006, SM 1976.2022.005, SM.1976.2022.019, VA01-1–3, VA06 (figures 18,19 and 19, table 10; electronic supplementary material, figure S3)

**Table 10.** Measurements of HBR_B2.3 tracks.

						digit length (DL)			DL ratios			divarication angles				
specimen	PG	L/R	L	W	L/W	II	III	IV	III/II	III/IV	III/L	II–III	III–IV	II–IV	te	M
GLAHM 114831	0.5	L*	15.85	n/a	n/a	n/a	12.08	13.05	n/a	n/a	n/a	n/a	n/a	n/a	n/a	n/a
GLAHM 152380	0.5	L*	19.54	13.46	1.45	10.79	13.78	13.02	1.28	1.06	70.52	31.73	21.65	53.38	7.77	0.58
2002.001-1	1	L*	14.54	12.00	1.21	7.20	11.23	7.91	1.56	1.42	77.26	31.39	34.59	65.98	6.93	0.58
2021.006	0.5	L*	17.58	15.42	1.14	n/a	13.68	10.00	n/a	1.37	77.82	n/a	n/a	72.35	9.10	0.59
2022.019	0.5	R*?	n/a	14.52	n/a	n/a	n/a	n/a	n/a	n/a	n/a	n/a	n/a	n/a	n/a	n/a
VA01-1	0.5	L*	17.52	12.79	1.37	8.54	11.52	13.74	1.35	0.84	65.75	30.12	34.35	64.47	5.19	0.41
VA01-2	0.5	R*?	15.65	11.74	1.33	7.13	13.18	10.86	1.85	1.21	84.22	20.28	23.33	43.61	6.10	0.52
VA01-3	0.5	L*	13.50	14.85	0.91	7.78	11.42	8.31	1.47	1.37	84.59	40.41	35.74	76.15	5.96	0.40
VA06	1	R*?	19.00	18.41	1.03	11.55	14.30	13.37	1.24	1.07	75.26	48.44	39.65	88.09	8.68	0.47
***average***	***0.6***	**—**	***16.65***	***14.15***	***1.21***	***8.83***	***12.65***	***11.28***	***1.46***	***1.19***	***76.49***	***33.73***	***31.55***	***66.29***	***7.10***	***0.51***

All lengths were measured in cm. The III/L ratio is expressed as a percentage. Divarication angles were measured in degrees. All track measurements were rounded to two decimal places.

#### 3.8.1. Diagnosis

Grouped under HBR_B2 due to small–medium track length ranges and hint of spindle-shaped digits. Metrically similar to HBR_B2.2 but distinguished by broader, rounded, subtly tapering digits lacking ungual marks. When present, sinuous and often incomplete slender inner digit margins are enclosed within a broad and round outer digit margin.

#### 3.8.2. Description

A small, asymmetric, tridactyl footprint, 13.5−19.5 cm long and 11.7−18.4 cm wide, with weak to sub-moderate l/w ratios (0.91−1.45) and relatively moderate mesaxony (0.40−0.59). Digits appear rounded and oval-shaped or vaguely spindle-shaped with poorly defined or absent phalangeal pads. Short, subtriangular ungual marks may be faintly distinguished or absent. Partial, sinuous, slender inner digit margins may be enclosed within a broad and round outer digit margin and may taper and indicate gracile ungual marks. Digit iii is often the longest digit and occupies >65% of the overall track length (average 76.3%). The average digit iii/ii ratio (1.46) is larger than the digit iii/iv equivalent (1.16). The average digit ii–iii interdigital angle (33.7°) is subequal to the digit iii–iv interdigital angle (31.6°).

#### 3.8.3. HBR_B2.3 material description

Unlike most morphotypes defined in this study, HBR_B2.3 is characterized by tracks with low anatomical fidelity (preservation grades <1, average 0.6) due to relatively featureless broad and rounded digit margins and absent or faint, subtriangular ungual marks. Occasional partial inner digit margins occur within these broad digits. HBR_B2.3 tracks were most likely registered in shallowly submerged palaeoenvironments due to a lack of radial/desiccation cracks emanating from tracks (*sensu* [44]).

At Valtos, HBR_B2.3 commonly occurs in a horizon of sandy limestone capping bed 5 (part of the lower Valtos Sandstone Formation), e.g. VA01-1−3 and VA06 (figure 18). Track-bearing surfaces are often featureless (occasionally rippled as is the case with VA06) with broken *Neomiodon* bivalves and cross-cutting calcite veins. VA01 contains five tracks, three complete (VA01-1−3) and two partial (VA01-4−5). The former three feature hints of tapering ungual marks and appear to bear a similar direction and may be associated. Interestingly, VA01-3 is the only track recorded from Valtos with a l/w ratio <1 (0.91), and features subtly separated lateral digits similar to some HBR_B2.2 tracks. VA06 differs with the presence of partial inner digit margins within rounder and broader digits and appear to taper toward triangular-shaped ungual marks—features absent on the outer round digit margins. Although these gracile inner digit margins are uncharacteristic of HBR_B2, the sub-medium track length which they occupy (19 cm), which is larger than any HBR_B3 track, cannot be ignored. The track is characterized by a subequal l/w ratio (1.03), weak mesaxony (0.47) and sub-right-angle digit ii–iv divarication (88.1°).

Examples from Lùb Score occur in two fine-grained sandstone horizons in the northeast edge of locality 1—the youngest at the locality. The broad outer digit margins are more spindle-shaped than those from Valtos and may feature phalangeal pads. These tracks often feature more clearly defined partial, sinuous inner digit margins within them, e.g. GLAHM 114831 and SM.1976.2022.019 (figure 19). Smaller tracks from this horizon can be distinguished as HBR_B3.2 when the inner digit margins are complete, visibly gracile and taper toward well-developed, sharp ungual marks (e.g. SM.1976.2022.010-1, see §3.10.3).

#### 3.8.4. HBR_B2 ichnotaxonomy

Morphotype HBR_B2.1 is distinguished from other morphotypes by its slenderer, spindle-shaped digits, digit ii–iv divarication angle range (30°−60°) and pronounced l/w ratio range (1.30−2.10). Early Jurassic *Eubrontes giganteus* tracks from North America also possess spindle-shaped digits, with moderate l/w ratios between approximately 1.40 and 1.50, a 67% digit iii/l ratio, and an average digit ii–iv divarication angle of 48°—all within HBR_B2.1 ranges [59,60]. Type *Eubrontes* material also possessed a pronounced l/w ratio (1.70) and moderate mesaxony (0.58) [60]. The moderately slender digits of some HBR_B2.1 tracks, e.g. VA10-7, 11 and SB02-1, also resemble *Changpeipus*—a similar ichnotaxon to *Eubrontes* [78,79]. Like HBR_B2.1, *Changpeipus* is characterized by moderate digit ii–iv divarication angles (50°) and pronounced digit iii/l ratios (63%) and l/w ratios (1.60) [79]. Despite these similarities, digit iv was shorter than digit ii in Middle Jurassic tracks from Morrocco referred to *Changpeipus* [80]—the opposite of HBR_B2.1. Additionally, the mesaxony of most HBR_B2.1 tracks is more pronounced (>0.50) than that of *Changpeipus* and many variants of *Eubrontes* [79]. Interestingly, the mesaxony of some HBR_B2.1 tracks overlaps with the 0.68 value of *Anchisauripus* [60]. However, the digits of *Anchisauripus* are not as consistently broad or moderately divaricated as most HBR_B2.1 tracks. Thus, although HBR_B2.1 exhibits metrics that overlap with several tridactyl ichnotaxa and a mixture of track characteristics, the morphotype is most similar to *Eubrontes* isp.

Like HBR_B2.1, HBR_B2.2 possesses small average track lengths (16.2 cm) and large digit iii/l ratios (73.4%) but a lower mesaxony (0.53) and spindle-shaped, tapering digits similar to *Eubrontes*. The wider average digit ii–iv divarication angle (69.4°) and weak (<1.30) l/w ratios of HBR_B2.2, however, contrast with *Eubrontes*. Such characters, combined with a ‘2 : 3 : 3’ phalangeal pad formula (when visible), are also reported in *Carmelopodus* tracks [26]. Despite this, HBR_B2.2 differs from *Carmelopodus* with respectively broader digits. The co-occurrence, association and morphological similarity (i.e. digit shape) of characteristic and referred HBR_B2.2 tracks (e.g. VA09-1 and 2) suggest that the morphotype subgroup possesses characters of *Eubrontes*, despite the observed variations.

HBR_B2.3 is also a small-sized track morphotype but differs with broader and rounder digits. Like HBR_B2.2, l/w ratios are weakly pronounced (0.91−1.45) but overlap into HBR_B2.1 ranges. Although rounded digits and lower l/w ratios are often associated with ornithopod trackmakers [55], the pes tracks lack manus counterparts and, although vague, often evidence features more strongly associated with theropod trackmakers including partial, slenderer inner digit margins within broad digits, with elongated triangular ungual marks. Like HBR_B1.2, HBR_B2.3 tracks variably suffer from erosion and other taphonomic processes and make ichnotaxon identification or referral difficult.

Overall, while HBR_B2.3 alone is not clearly consistent with a named ichnotaxon, HBR_B2.1 possesses clearer metrics and morphologies which align well with *Eubrontes*. These characteristics includes spindle-shaped, moderately slender digits, an average moderate l/w ratio (1.58), 72.2% digit iii/l ratio and 47.4° digit ii–iv divarication angle. Although the metrics of HBR_B2.2 are inconsistent with *Eubrontes*, i.e. moderate mesaxony (0.48–0.59), weak l/w ratios ≤1.30 (1.04−1.30) and wide digit ii–iv divarication >60° (average 69.4°), the subgroup morphological characters (i.e. digit shape) most resemble *Eubrontes*.

### 3.9. HBR_B3.1

**Figure 20.**
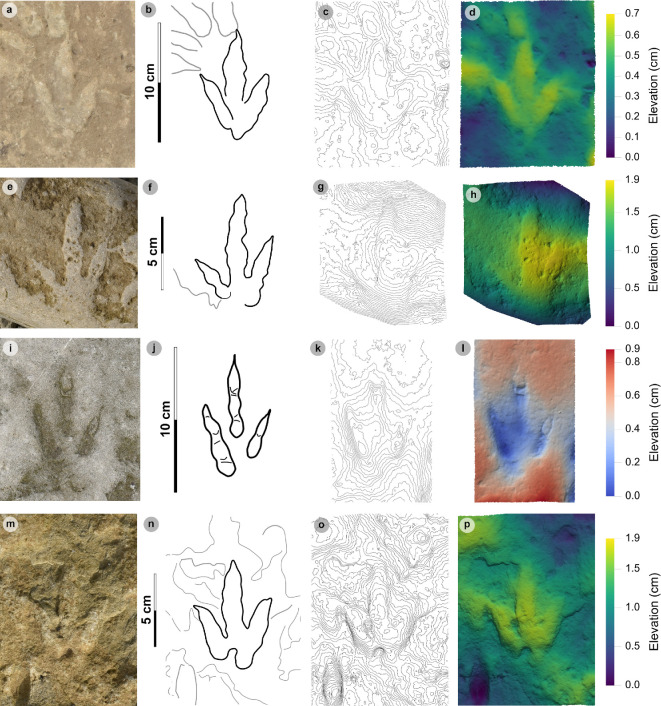
Photographic and digital representations of HBR_B3.1 tracks from Lùb Score. From left to right, photograph, outline, 0.5 mm contour map and DEM of: (a–d) SM.1976.2006.021-1, (e–h) SM.1976.2024.002, (i–l) SM.1976.2022.012-1 and (m–p) SB02-4. The tracks characterize the morphotype with pronounced l/w ratios and narrow digit ii–iv divarication angles. SM.1976.2006.021-1 and SM.1976.2024.002 were registered in a poorly consolidated substrate and, due to modern erosion, are surface worn. The oval-shaped, longer than wide phalangeal pads of SM.1976.2022.012-1 are indicated by hollows or sediment infill within digits. SB02-4 occurs alongside two other HBR_B2.1 tracks.

**Figure 21.**
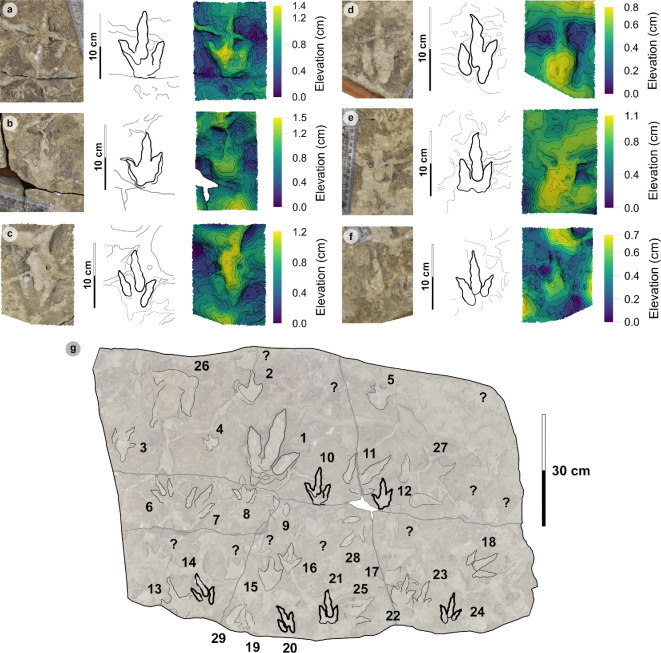
HBR_B3.1 tracks present on SM 1976.2002.007. Photograph or textured orthomosaic, outline with scale and 1 mm contoured DEM of (a) SM 1976.2002.007-10, (b) 12, (c) 14, (d) 20, (e) 21 and (f) 24. Many of the tracks on SM 1976.2002.007 are surface worn, positive relief impressions composed of a fine-grained sandstone which originally infilled a siltstone with desiccation cracks. (g) Outline of SM 1976.2002.007 overlaid onto textured orthomosaic with tracks numbered. The figured HBR_B3.1 tracks are bold. The HBR_B3.1 tracks appear to be directionally associated with similar HBR_B3.2 tracks, e.g. tracks 6 (HBR_B3.2) and 14 (HBR_B3.1) form a track association.

**Figure 22.**
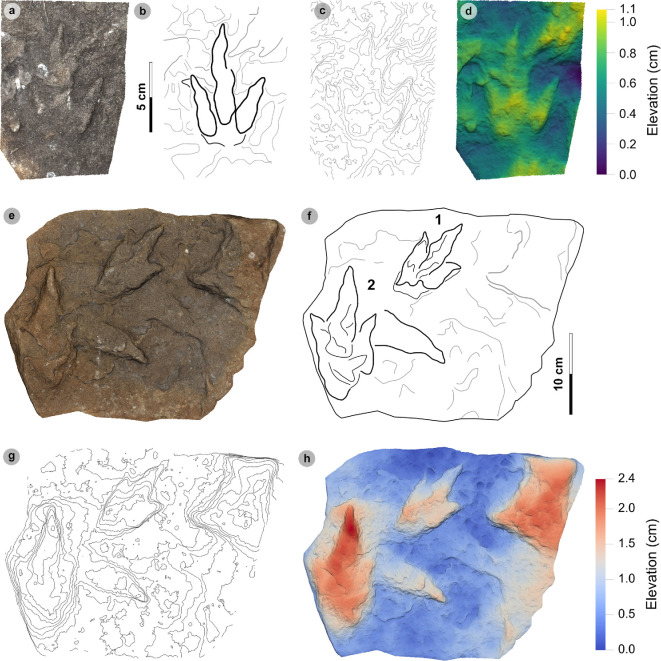
Digital representations of HBR_B3.1 tracks from the upper Valtos Sandstone Formation. (a) Textured orthomosaic, (b) outline, (c) 1 mm contour map and (d) DEM of SM.1976.2006.003-2. Note that the track is part of a larger slab. (e) Textured orthomosaic, (f) outline, (g) 2 mm contour map and (h) DEM of SM.1976.2006.009-1−2. Track 1 is 2.7 cm shorter in length than track 2.

**Figure 23.**
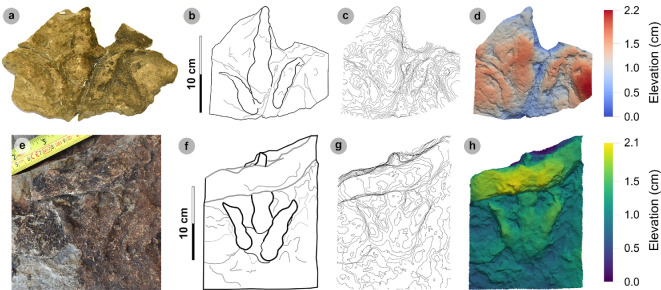
Photographic and digital representations of HBR_B3.1 tracks from the lower Valtos Sandstone Formation. Photograph, outline, 1 mm contour map and DEM of (a–d) GLAHM 114833 and (e*–*h) VA15. Both tracks are composed of fine-grained sandstone. Digit iii on VA15 is abruptly intersected by a large desiccation crack.

**Material**: GLAHM 114833, SM.1976.2002.006-1, SM 1976.2002.007-10, 12, 14, 20, 21, 24, SM.1976.2006.003-2, SM.1976.2006.009-1–2, SM.1976.2006.021-1, SM.1976.2022.012-1, SB02-4, VA15 (figures 20–23, table 11)

**Table 11.** Measurements of HBR_B3.1 tracks.

						digit length (DL)			DL ratios			divarication angles				
specimen	PG	L/R	L	W	L/W	II	III	IV	III/II	III/IV	III/L	II–III	III–IV	II–IV	te	M
GLAHM 114833	1	L*	13.30	7.76	1.71	5.51	12.00	5.85	2.18	2.05	90.23	22.37	26.60	48.97	7.64	0.98
2002.006-1	1	L*	7.51	4.58	1.64	3.05	5.33	3.62	1.75	1.47	70.97	28.95	25.81	54.76	3.53	0.77
2002.007-10	1.5	R*	8.68	5.68	1.53	3.56	6.26	4.81	1.76	1.30	72.12	32.43	25.70	58.13	4.14	0.73
2002.007-12	1	L*	6.55	4.28	1.53	n/a	4.61	n/a	n/a	n/a	70.38	n/a	n/a	n/a	2.51	0.59
2002.007-14	1	L*	8.00	4.97	1.61	3.50	5.61	4.34	1.60	1.29	70.13	16.04	33.30	49.34	3.64	0.73
2002.007-20	1	L*	7.37	3.39	2.17	3.27	5.31	4.36	1.62	1.22	72.05	7.12	19.40	26.52	3.12	0.92
2002.007 -21	1	L*	8.52	4.02	2.12	4.05	6.03	5.04	1.49	1.20	70.77	16.58	20.39	36.97	3.53	0.88
2002.007-24	1	R*	8.93	4.19	2.13	4.45	5.50	5.62	1.24	0.98	61.59	16.55	19.97	36.52	3.32	0.79
2006.003 -2	1.5	R*	7.22	4.58	1.58	3.59	6.87	3.97	1.91	1.73	95.15	16.11	25.90	42.01	3.47	0.76
2006.009-1	1.5	R*	9.40	5.23	1.80	4.77	7.13	6.27	1.49	1.14	75.85	20.14	5.07	25.21	3.98	0.76
2006.009-2	1	R*	12.09	6.15	1.97	5.71	8.97	8.44	1.57	1.06	74.19	33.25	12.14	45.39	5.31	0.86
2006.021-1	1	L*	7.93	4.88	1.63	4.32	6.15	5.50	1.42	1.12	77.55	26.82	24.36	51.18	3.44	0.70
2022.012 -1	2	L	7.07	4.02	1.76	2.88	4.49	4.34	1.56	1.03	63.51	19.45	24.51	43.96	3.14	0.78
2024.002 -1	1.5	L*	6.88	4.57	1.51	2.52	n/a	4.36	n/a	n/a	n/a	40.97	17.45	58.42	3.53	0.77
SB02-4	1.5	L*	6.92	4.54	1.52	3.79	5.47	5.06	1.44	1.08	79.05	21.50	23.71	45.21	2.43	0.54
VA15	1.5	L*	6.67	3.87	1.72	3.03	4.79	3.86	1.58	1.24	71.81	18.79	26.22	45.01	3.06	0.79
***average***	***1.25***	**—**	***8.32***	***4.79***	***1.75***	***3.87***	***6.30***	***5.03***	***1.62***	***1.28***	***74.36***	***22.47***	***22.04***	***44.51***	***3.74***	***0.77***

All lengths were measured in cm. The III/L ratio is expressed as a percentage. Divarication angles were measured in degrees. All track measurements were rounded to two decimal places.

**Referred material:** SM 1976.2002.007-7, 8, 22, 23, SM 1976.2006.017a, SM 1976.2006.021-3, SM 1976.2007.002, SM 1976.2009.003-2, SM 1976.2011.001-2, SM 1976.2016.001b-3, SM 1976.2022.010-2, SM.1976.2022.026, SM.1976.2024.003-1, VA09-4, 9, VA10-26, SB02-3 (electronic supplementary material, table S4)

#### 3.9.1. Diagnosis

HBR_B3 tracks feature gracile, tapering digits and elongated, narrow ungual marks. HBR_B3.1 tracks are <15 cm in length and distinguished from HBR_B3.2 with at least two of the following metrics: pronounced l/w ratios >1.50, mesaxonic indices >0.7, or digit ii–iv divarication angles <50°. Digit iii is pronounced and generally >70% of the track length. Morphologically, digits are often poorly separated.

#### 3.9.2. Description

Tiny to small, asymmetric, tridactyl footprint of 6.6−13.3 cm in length and 3.4−7.8 cm wide. Tracks possess moderate-pronounced l/w ratio (1.51−2.17) and mesaxony (0.54−0.98) ranges. Digits are gracile, and taper toward sharp, elongated, narrow ungual marks. Digits are rarely well-separated. Digit iii is consistently the longest digit and occupies on average 74.4% of the total track length. Digit iii/ii ratios are larger (average 1.62) than digit iii/iv equivalents (average 1.28). Phalangeal pads are longer than wide and oval-shaped based on digit margins and are narrower than other morphotypes. The digit ii–iv divarication angle varies between 25.2° and 58.4° (average 44.5°).

#### 3.9.3. HBR_B3.1 material description

HBR_B3.1 is considered a ‘narrow’ form variant of HBR_B3 and is defined from 16 tracks with preservation grades >1. The ‘narrow’ label is applied as HBR_B3.1 tracks are narrowly divaricated (average 44.5°) with pronounced l/w ratios (average 1.75) and mesaxony (0.77). Indeed, the ranges of these metrics generally exceed those of HBR_B3.2 (see §3.10.3). If lumped together with HBR_B3.2, the metrical nuance of HBR_B3 would not allow the morphotype to be as distinguishable.

HBR_B3.1 occurs most commonly at Lùb Score as surface-worn impressions from undetermined horizons of poorly laminated and consolidated fine-grained sandstone, e.g. SM.1976.2006.021-1 and SM.1976.2024.002 (figure 20a–h). As a result, digit margins and phalangeal pads can appear worn. Such features, however, are more sharply defined in tracks with relief, including SM.1976.2022.012-1 (figure 20i–l). The negative relief track features clear oval-shaped phalangeal pads (emphasized by sediment infill or hollow relief) and ungual marks. Although the lateral digits appear subtly separated from the posterior margins of digit iii similar to some HBR_B3.2 tracks, SM.1976.2022.012-1 features HBR_B3.1 morphologies. These include pronounced l/w ratios (1.76) and mesaxony (0.78), and narrow digit ii–iv divarication angle (44.0°). SB02-4, a positive relief track, is also sharply defined (figure 20m–p). The track occurs on the same surface as a referred HBR_B3.1 track (SB02-3) and faces the opposite direction to two HBR_B2.1 tracks (SB02-1−2) (figure 12). Although the track’s l/w ratio is at the lower end of the HBR_B3.1 range (1.52), the track features clearer HBR_B3.1 characteristics including a weak digit ii–iv divarication angle (45.2°).

The most notable association of HBR_B3.1 tracks occurs on the surface of SM 1976.2002.007—which contains at least 29 tracks representing multiple morphotypes registered in a similar timeframe (figure 21). The tracks are preserved as worn positive relief casts composed of fine-grained sandstone, which infilled a siltstone—the original track-bearing horizon [24]. Tracks 12, 14, 20, 21 and 24 feature relatively distinguishable digit margins and are used to characterize HBR_B3.1 (figure 21a–f). The tracks possess the following HBR_B3.1 characters: pronounced l/w ratios (1.53−2.17) and mesaxony (0.59−0.92), and predominantly narrow divarication angles (26.5°−58.1°). Some HBR_B3.2 tracks form track associations with HBR_B3.1 tracks, e.g. tracks 6 and 14—suggesting both morphotypes could be registered by the same trackmaker. This hypothesis is further emphasized by most tracks sharing a southwest bearing [24]. This directional association is additionally shared by track 1, a single HBR_B2.1 track. Collectively, the tracks were hypothesized by [24] to represent post-hatching care (see §4.3).

At Valtos, HBR_B3.1 is scarcer and typically known from positive relief impressions in isolated slabs. The best preserved are composed of medium-grained sandstones from the upper Valtos Sandstone Formation (e.g. SM.1976.2006.003-2 and SM.1976.2006.009-1−2) (figure 22). Based on its bulged digit margins, SM.1976.2006.009-1−2 feature longer than wide, oval shaped phalangeal pads. The exact pad configurations of either are uncertain due to worn digit margins (at least two distal phalangeal pads can be distinguished on digits ii–iii). Despite this, both tracks possess narrow <60° divarication angles (track 1 = 25.2°, track 2 = 45.4°) and pronounced mesaxony (track 1 = 0.76, track 2 = 0.86). Similar tracks are recorded in the lower unit, e.g. GLAHM 114833 (49.0° divarication angle, 0.98 mesaxony) and VA15 (45.0° divarication angle, 0.79 mesaxony) (figure 23). Referred examples of HBR_B3.1, such as VA09-4 and 9, occur amongst diagnosable HBR_B2.1 and 2.2 tracks in bed 25 (figure 17). A third track referred to HBR_B3.1 from bed 25, VA10-26, is partially overprinted by a HBR_B2.2 referred track (figure 14).

### 3.10. HBR_B3.2

**Figure 24.**
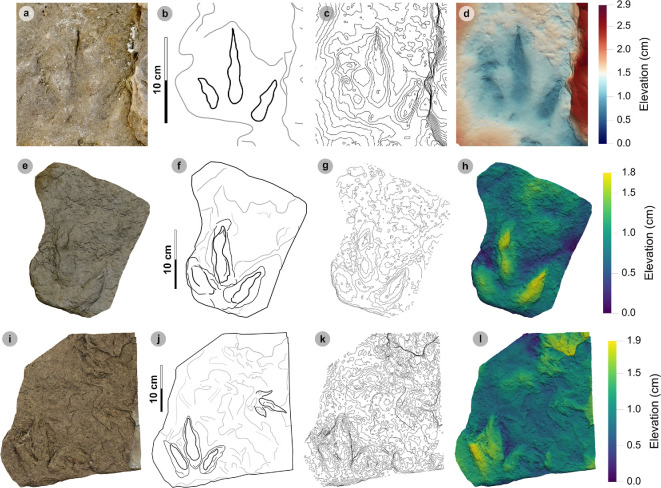
Digital representations of HBR_B3.2 tracks from Lùb Score. Textured orthomosaic, outline, contour map and DEM of (a–d) SM.1976.2023.004-1, (e–h) SM.1976.2022.014 and (i–l) SM.1976.2022.010-1. Note that the contours on the contour map for SM.1976.2022.014 are separated by 2 mm, and 1 mm for SM.1976.2022.010-1 and SM.1976.2023.004-1. SM.1976.2022.010-1 and SM.1976.2022.014 possess broad outer digit margins which alone results in a resemblance to HBR_B2.2. The tracks were measured and classified under HBR_B3.2 based on their well-separated, sharply defined gracile inner margins, which taper toward sharp, narrow ungual marks.

**Figure 25.**
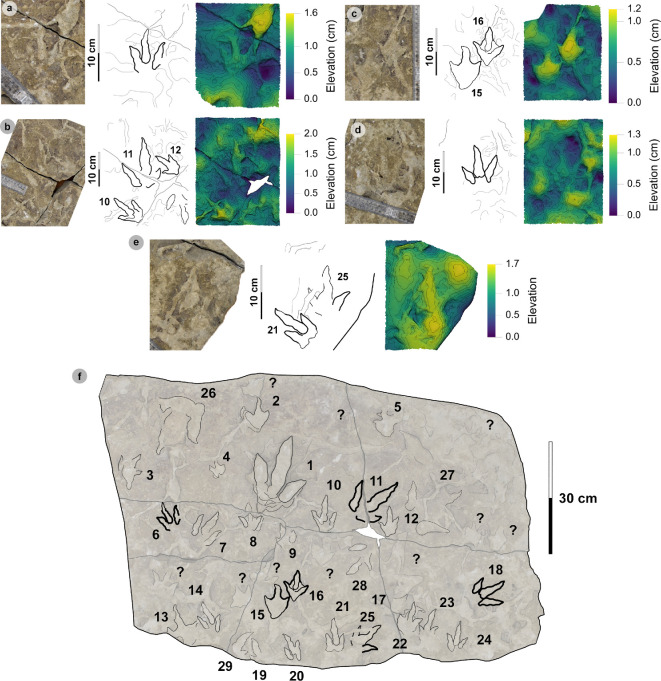
HBR_B3.2 tracks present on SM 1976.2002.007. Photograph, outline with scale and 1 mm contoured DEM for: (a) SM 1976.2002.007-6, (b) 11, (c) 15−16, (d) 18 and (e) 25. Note that track 11 is surrounded by HBR_B3.1 tracks 10 and 12, and track 25 is opposite track 21 (a HBR_B3.1 track). The tracks are surface worn positive relief impressions composed of a fine-grained sandstone which originally infilled a siltstone with desiccation cracks. (f) Outline of SM 1976.2002.007 overlaid onto a textured orthomosaic with tracks numbered. The figured diagnostic and referred HBR_B3.2 tracks are bold. The HBR_B3.2 diagnosed tracks share the same gracile digits and narrow ungual marks with HBR_B3.1. The tracks furthermore appear to be directionally associated with HBR_B3.1 equivalents.

**Figure 26.**
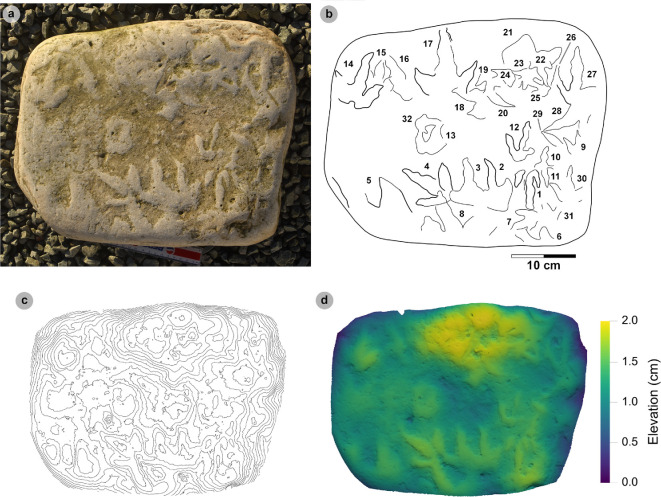
Track cluster with associated referred HBR_B3 tracks. (a) Photograph, (b) outline with track numbers, (c) 1 mm contour map and (d) DEM of SM.1976.2024.003. The surface features 32 tracks—track 1 is referred to HBR_B3.1, while tracks 4, 12, 14 and 17 are referred to HBR_B3.2. Many of the other tracks are too heavily surface worn and incomplete to be classified. Despite this, the surface shows multiple HBR_B3 sized tracks bearing multiple directions.

**Figure 27.**
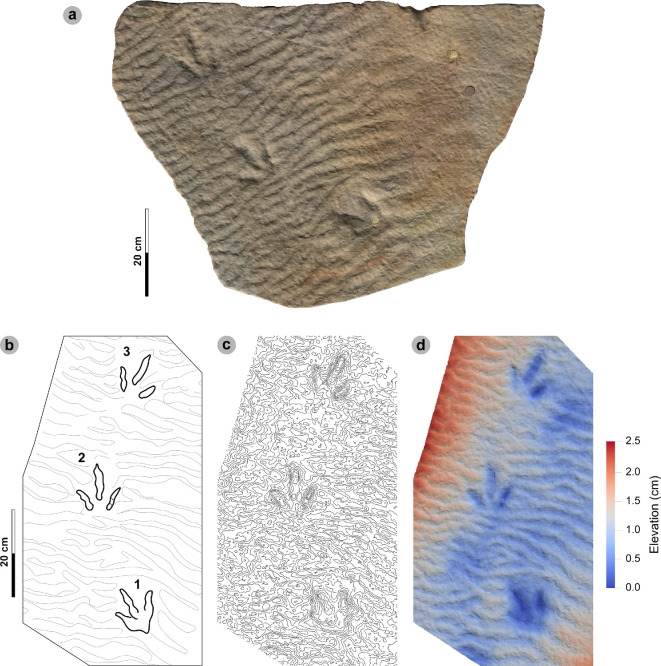
Digital representations of SM.1976.2004.002a. The tracks were most likely registered in a shallowly submerged substrate—indicated by the absence of radial and desiccation cracks and proliferation of ripples. Although the outermost digit margins are rounded, the gracile inner margins (the most sharply defined), weak l/w ratios (1.19−1.22), moderate mesaxony (0.43−0.56) and well separated, gracile inner digit margins suggest a HBR_B3.2 diagnosis.

**Figure 28.**
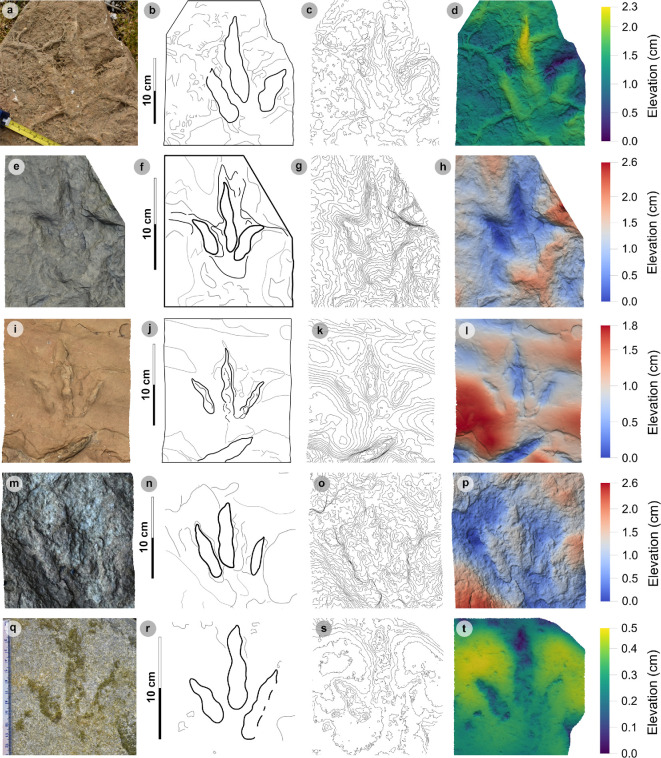
Photographic and digital representations of HBR_B3.2 tracks from Valtos. Photograph or textured orthomosaic, outline, contour map, and DEM of: (a–d) SM.1976.2002.005, (e–h) SM.1976.2022.018b, (i–l) SM.1976.2018.001-1, (m–p) VA10-22 and (q–t) SM.1976.2024.004. Note that the contours are spaced by 2 mm for SM.1976.2002.005, 1 mm for SM.1976.2022.018b, SM.1976.2018.001-1, VA10-22 and 0.5 mm for SM.1976.2024.004. All tracks are characterized by low l/w ratios, moderate mesaxony and wide digit ii–iv divarication angles.

**Material**: GLAHM 114904, SM.1976.2002.005, SM 1976.2002.007-6 and 18, SM.1976.2004.002a-1–3, SM.1976.2006.006-2, SM.1976.2009.001, SM.1976.2018.001-1, SM.1976.2022.010-1, SM.1976.2022.014, SM.1976.2022.018b, SM.1976.2022.021, SM.1976.2023.003-1, SM.1976.2023.004-1, SM.1976.2024.004, VA10-22 (figures 24–28, tables 12–14; electronic supplementary material, figure S4)

**Table 12.** Measurements of HBR_B3.2 tracks.

						digit length (DL)			DL ratios			divarication angles				
specimen	PG	L/R	L	W	L/W	II	III	IV	III/II	III/IV	III/L	II–III	III–IV	II–IV	te	M
GLAHM 114904	1	R*	6.82	5.41	1.26	3.03	4.78	5.07	1.58	0.94	70.09	29.68	30.20	59.88	2.86	0.53
2002.005	1	R*	13.10	10.19	1.29	5.94	10.43	7.85	1.76	1.33	79.62	35.80	15.81	51.61	6.20	0.61
2002.007-6	1	R*	6.81	4.93	1.38	3.05	5.16	3.70	1.69	1.39	75.77	17.67	23.44	41.11	3.19	0.65
2002.007-18	1	L*	9.13	6.51	1.40	3.25	6.98	4.88	2.15	1.43	76.45	33.91	36.98	70.89	5.08	0.78
2004.002a-1	1	R	9.82	8.28	1.19	5.90	8.07	5.20	1.37	1.55	82.18	32.22	29.03	61.25	3.52	0.43
2004.002a-2	1	L	10.77	8.80	1.22	5.62	7.74	5.60	1.38	1.38	71.87	28.39	36.62	65.01	4.79	0.54
2004.002a-3	1.5	R	10.25	8.61	1.19	5.90	7.38	5.20	1.25	1.42	72.00	28.09	29.66	57.75	4.79	0.56
2006.006-2	1	L*	9.77	9.01	1.08	4.14	7.00	5.18	1.69	1.35	71.65	47.84	59.95	107.79	6.34	0.70
2009.001	1	R	11.55	8.85	1.31	5.65	9.05	5.36	1.60	1.69	78.35	12.96	33.78	46.74	5.20	0.59
2018.001-1	2	R	7.59	7.24	1.05	3.75	7.31	4.25	1.95	1.72	96.31	25.16	31.97	57.13	3.30	0.46
2022.010-1	2	R*	12.09	10.44	1.16	5.99	10.88	7.46	1.82	1.46	89.99	27.69	43.97	71.66	5.39	0.52
2022.014	1.5	L*	12.40	10.41	1.19	6.02	8.75	6.20	1.45	1.41	70.56	36.22	34.69	70.91	6.42	0.62
2022.018b	2	R	8.19	6.03	1.36	4.09	6.42	4.13	1.57	1.55	78.39	36.49	26.97	63.46	4.64	0.77
2022.021	1	L	6.40	5.55	1.15	2.74	5.17	3.61	1.89	1.43	80.78	29.25	26.35	55.60	3.03	0.55
2023.003-1	1.5	L*	10.90	8.04	1.36	4.34	6.57	7.37	1.51	0.89	60.28	27.42	35.37	62.79	4.85	0.60
2023.004-1	1.5	R	9.30	8.54	1.09	3.81	7.25	4.75	1.90	1.53	77.96	24.18	29.45	53.63	4.62	0.54
2024.004	1	R	10.71	7.57	1.41	4.09	7.80	6.79	1.91	1.15	72.83	31.43	26.28	57.71	5.05	0.67
VA10-22	1	R*	7.98	7.67	1.04	3.92	7.00	5.74	1.79	1.22	87.72	19.42	20.71	40.13	3.18	0.41
***average***	***1.28***	**—**	***9.64***	***7.89***	***1.23***	***4.51***	***7.43***	***5.46***	***1.68***	***1.38***	***77.38***	***29.10***	***31.74***	***60.84***	***4.58***	***0.58***

All lengths were measured in cm. The III/L ratio is expressed as a percentage. Divarication angles were measured in degrees. All track measurements were rounded to two decimal places.

**Table 13.** Measurements for the SM.1976.2004.002a trackway.

specimen	L	h	P	λ	WAP	α	γ
2004.002a-1	9.82	0.39	0.2712	0.5225	7.95	17.07	n/a
2004.002a-2	10.77	0.43	0.2751	n/a	n/a	16.83	146.11
2004.002a-3	10.25	0.41	n/a	n/a	n/a	n/a	n/a
***average***	***10.28***	***0.41***	***0.27***	***n/a***	***n/a***	***16.95***	***n/a***

All values were rounded to two decimal places. Total track length (L) and width of pes angulation (WAP) were measured in cm. Hip height (h), pace (P) and stride (λ) lengths were measured in m. Angles of rotation (α) and pace angulation (γ) were measured in degrees.

**Referred material**: GLAHM 152986, SM 1976.2002.007-11, 15, 16, 25, SM 1976.2006.001, SM.1976.2006.20, SM 1976.2006.021-2, SM 1976.2006.021-4, SM 1976.2021.005, SM 1976.2021.007-2–3, SM.1976.2006.009, SM 1976.2022.015-1–2, SM.1976.2022.020-1–2, SM.1976.2023.003-2, SM.1976.2024.003-4, 14, 17, VA13-2–3, SB01 (electronic supplementary material, table S5)

#### 3.10.1. Diagnosis

Diagnosed under HBR_B3 as tracks feature gracile, tapering digits with indications of oval-shaped phalangeal pads (based on digit margins) and elongated, narrow ungual marks. Differentiated from morphotype HBR_B3.1 with at least two of the following: weak to moderate l/w ratios (<1.40), mesaxonic indices typically <0.7 and digit ii–iv divarication angles usually >50°. Morphologically, digits are often well separated.

#### 3.10.2. Description

Tiny to small asymmetric, tridactyl footprint, 6.4−13.1 cm long and 4.9−10.4 cm wide, with generally weak l/w ratios (1.04−1.40) and sub-moderate to pronounced mesaxonic indices typically <0.7 (0.41−0.78). Digits are slender, and taper toward pronounced elongated, triangular shaped ungual marks. Digit iii is often the longest digit and occupies on average 77.4% of the total track length. When visible, tracks can possess a phalangeal pad formula of ‘2 : 3 : 3’. Digit iii/ii ratios (average 1.68) exceed digit iii/iv equivalents (average 1.38). The digit ii–iv divarication angle ranges between 40.1° and 107.8° (average 60.8°).

#### 3.10.3. HBR_B3.2 material description

Morphotype HBR_B3.2 is considered a ‘wide’ form variant of HBR_B3 and most frequently observed as isolated occurrences in well-indurated, fine-grained sandstones at Lùb Score. The subgroup is defined from 18 tracks with preservation grades >1. The ‘wide’ label is applied as HBR_B3.2 tracks are generally more widely divaricated (average 60.8°) with weaker l/w ratios (average 1.23) and less pronounced mesaxony (average 0.58) than HBR_B3.1.

Negative relief specimens often feature more well-separated digits than HBR_B3.1 with sharp ungual marks and distinguishable phalangeal pad margins (e.g. SM.1976.2023.004-1) (figure 24a–d). SM.1976.2023.004-1 features a pronounced anteromedially oriented digit iii ungual mark which can create the illusion of pronounced mesaxony and classification under HBR_B3.1. Such marks however are highly variable in length and are not included in track measurements. SM.1976.2023.004-1 is differentiated from HBR_B3.1 with a weaker l/w ratio (1.09) and moderate mesaxony (0.54) (table 12).

Some HBR_B3.2 tracks from the fine-grained sandstones in the northeastern corner of locality 1 can feature broad outer digit margins and sharp, gracile inner margins within them (e.g. SM.1976.2022.014 and SM.1976.2022.010-1) (figure 24e–l). This can create the illusion a track was imparted by a larger animal and resemble morphotypes such as HBR_B2. However, the presence of gracile inner margins with sharp ungual marks characterize these tracks under HBR_B3. The tracks possess HBR_B3.2 metrics including weak l/w ratios (1.16−1.19), mesaxony <0.7 (0.52−0.62) and pronounced digit ii–iv divarication angles (70.9°−71.7°).

Although generally found isolated in individual slabs, examples on SM 1976.2002.007 including tracks 6 and 18 (and referred tracks 11, 15, 16, 25) are directionally associated with both diagnostic and referred HBR_B3.1 tracks, e.g. track 6 (HBR_B3.2) with track 14 (HBR_B3.1), and were likely impressed by the same trackmakers (figure 25). Tracks 6 and 18 are characterized under HBR_B3.2 by weak l/w ratios (1.38−1.40) and one of the two characteristics: mesaxony <0.7 (0.65 on track 6) and large divarication angle (70.9° on track 18). Further associated examples of referred tracks attributed to either HBR_B3 subgroup occur on SM.1976.2024.003 and bear multiple directions (figure 26). Tracks 4 and 14 feature weak l/w ratios (1.02−1.23), mesaxony <0.7 (0.49−0.60) and pronounced divarication angles (65.4°−84.8°).

Although trackways composed of these morphotypes are hard to distinguish on either surface and generally rare, specimen SM.1976.2004.002a provides insight into the gait deployed by a small trackmaker capable of registering HBR_B3 tracks (figure 27). SM.1976.2004.002a consists of a single stride (three negative relief tracks) across a rippled surface composed of a poorly laminated, fine-grained sandstone. Given the short distance between ripple crests (average 3.5 cm), lack of desiccation cracks and vague ripple intrusions crosscutting the tracks, the trackway was likely imparted into a shallowly submerged substrate. This may explain why the outermost digit margins appear subtly rounded (*sensu* [44]). Despite this, present HBR_B3.2 characteristics include weak l/w ratios (1.19−1.22), moderate mesaxony (0.43−0.56), and well-separated, gracile inner digit margins. The trackmaker maintained a consistent pace of 27.1−27.5 cm and strode 52.3 cm, with shallow angles of rotation (16.8°−17.1°) and obtuse pace angulation (146.1°) (table 13). Based on an average track length of 10.3 cm, the trackmaker hip height is estimated at approximately 41 cm. Over this single stride, the trackmaker deployed an estimated walking gait of 1.26 and velocity of 0.67−0.74 m s^−1^ (2.41−2.66 km h^−1^ = 1.50–1.65 mph).

**Table 14.** Gait and velocity measurements for the SM.1976.2004.002a trackway.

Track λ	L / R	L	h	λ	λ/h	V (A)	V (RT)
2004.002 a-1−3	R	10.28	0.41	0.52	1.26	0.67	0.74

All values were rounded to two decimal places. Track length (L) represents the trackway average and was measured in cm. Hip height (h) and stride length (λ) were measured in m. Velocity was measured in m s^−1^. ‘V (A)’ is measured according to [62] and ‘V (RT)’ according to [63].

In the Valtos Sandstone Formation, HBR_B3.2 is rare and generally known from isolated occurrences in the well-indurated, fine-grained sandstones from the lower unit, e.g. SM.1976.2002.005 and SM 1976.2022.018b (figure 28a–h). Both tracks are characterized under HBR_B3.2 with weak l/w ratios (1.29−1.36) and one of the two characteristics: mesaxony <0.7 (0.61 on SM.1976.2002.005) and large divarication angle (63.5° on SM 1976.2022.018b). A desiccation crack which extends out of digit iii on SM.1976.2002.005 can create the illusion of more pronounced track length (ungual mark was omitted from measurements). The most sharply defined track, SM.1976.2018.001-1, is in negative relief and features distinctive oval-shaped concave phalangeal pad impressions and vague interdigital creases (with a probable ‘2 : 3 : 3’ phalangeal pad formula) (figure 28i–l). Despite variable mesaxonic indices, which overlap into HBR_B3.1 ranges (up to 0.77 in SM.1976.2022.018b), the tracks possess weak l/w ratios (<1.30) and divarication angles >50°—within the ranges of HBR_B3.2 (table 12).

Although the three ascribed HBR_B3.2 tracks are preserved in fine-grained sandstones, the track-bearing surfaces of each differ. The surface of SM.1976.2002.005 likely desiccated through subaerial exposure after track registration. In contrast, we can be more confident that SM.1976.2018.001-1 was registered on a submerged surface as the bedding plane preserves ripples and lacks any desiccation cracks emanating from the track to suggest subaerial exposure. The surface of SM.1976.2022.018b is freshly exposed and featureless.

Two HBR_B3.2 tracks are recorded in limestones and include VA10-22 and SM.1976.2024.004 (figure 28m–t). VA10-22 is the only characterizable HBR_B3.2 track from the medium- to coarse-grained sandy limestone of bed 25 (figure 28m–p). This is based on its well-separated, tapering gracile digits, subequal l/w ratio (1.04) similar to SM.1976.2018.001-1, and weak mesaxony (0.41). The track faces the opposite direction to VA10-1—large-sized HBR_B1.4 track (figure 8m). SM.1976.2024.004 is presently the only track recorded in a shelly limestone from Port Earlish. Despite its worn gracile digit margins, the track features a faint ‘2 : 3 : 3’ phalangeal pad formula (figure 28q–t). Like VA10-22, the lateral digits are separated from the posterior margin of digit iii.

From the medium-grained sandstones of the upper Valtos Sandstone Formation, SM.1976.2006.006-2 is unusually widely divaricated between digits ii–iv at 107.8°—the largest divarication angle recorded at Valtos—and faces the opposite direction to a HBR_B2.2 track (figure 16).

#### 3.10.4. HBR_B3 ichnotaxonomy

Despite their distinctions, both HBR_B3 subgroups possess multiple overlapping morphologies, e.g. gracile digits tapering toward narrow, elongated ungual marks and longer than wide, oval-shaped phalangeal pads when visible. Although one HBR_B3 subgroup track may be distinguished by at least two metrics, particularly l/w ratio, mesaxony and digit ii–iv divarication angle, one or more of these may overlap with a track of the other subgroup. Additionally, both subgroups occur together in track associations, e.g. tracks 6 and 14, and tracks 15 and 8 on SM 1976.2002.007. For these reasons, we suggest both HBR_B3 subgroups closely correspond to each other, despite the minor but defined differences between them, and may have been made by the same trackmaker, or two very similar trackmakers with similar foot morphologies or kinematics. The distinction of each HBR_B3 subgroup allows metrical nuance to be more clearly conveyed than as a single morphotype with broad ranged metrics. Indeed, the metrical and morphological variation of either subgroup is consistent with multiple ichnotaxa. We discuss these distinctions as follows.

The smaller <15 cm track lengths, pronounced average l/w ratios (1.75) and mesaxony (0.77) and narrower digit ii–iv divarication angles (44.5°) of morphotype HBR_B3.1 are most similar to *Grallator* [59]. This concurs with previous assessments by [24]. Early Jurassic North American specimens of this ichnotaxon tend to possess more extreme l/w ratios (2.64) and mesaxony (1.22) than those observed in HBR_B3.1 [60]. Late Jurassic *Grallator* isp. tracks from Spain possess pronounced l/w ratios (1.73−2.50) and low digit ii–iv divarication angles (32°−55°) [81]. These track measurements overlap with morphotype HBR_B3.1 ranges despite their larger track length ranging up to 21.5 cm and inclusion of ungual marks (which, unlike the Skye tracks, appear consistently preserved between tracks). A similar tiny tridactyl track with a l/w ratio of 1.75 (BP3_13) was recorded in the Lealt Shale Formation by [21]. Using the Hebridean series, this track belongs to the HBR_B3.1 morphotype.

Although its digits are similarly gracile to HBR_B3.1, HBR_B3.2 is, on average, more widely divaricated (60.8°) with less pronounced l/w ratios (often <1.30, average 1.23) and mesaxony (average 0.58). These characteristics, in combination with the occasional impression of a ‘2 : 3 : 3’ phalangeal pad formula and well-separated digits, are reminiscent of *Carmelopodus. Carmelopodus* is characterized by ‘well developed’ distal ungual marks and weak l/w ratios [26]. These similarities are particularly evident in tracks including SM.1976.2018.001-1, which closely resembles North American type material [26]. Other HBR_B3.2 shared characteristics, including digit gracility and stricter moderately pronounced mesaxony (0.55−0.67), are of particular interest, as these characters overlap with HBR_B3.1 tracks similar to *Grallator*. These overlapping characteristics are further emphasized by the two morphotype subgroups occurring on the same track-bearing surfaces as track associations, e.g. SM 1976.2002.007-6 and 14 as reported by [24]. This likely suggests tracks 6 and 14 were registered by the same individual trackmaker (figures 21,25 and 25).

Other Middle Jurassic *Carmelopodus* tracks can possess characteristics which overlap with either one of our HBR_B3 subgroups. For example, Middle Jurassic *Carmelopodus* tracks from the Bingtu tracksite in China possess a ‘2 : 3 : 3’ phalangeal pad formula and an average 1.30 l/w ratio which unequivocally falls within HBR_B3.2 ranges [82]. The 0.62 mesaxony, however, overlaps with both HBR_B3 subgroups. Despite this, the digits of the Bingtu tracks are well-separated, as seen in most HBR_B3.2 examples from Lùb Score, e.g. SM.1976.2022.10-1 and SM.1976.2023.004-1. A single *Carmelopodus* track presumed to originate from the Bathonian aged Forest Marble of Buckinghamshire, England, also possesses characters of both HBR_B3 subgroups: well-separated, gracile digits and a ‘2 : 3 : 3’ phalangeal pad formula as per HBR_B3.2; and a 0.67 mesaxony—within the range of both HBR_B3 subgroups [15,26]. Further Middle Jurassic *Carmelopodus* tracks from Morocco possess moderate l/w ratios (1.60), mesaxonic indices (0.59) and digit ii–iv divarication angles (approx. 53°) which overlap with both Hebridean HBR_B3 subgroups [80]. The l/w ratios of the Moroccan tracks are much larger than those in North American tracks (average reported as 1.18 by [26]) and more closely fit in with HBR_B3.1 ranges. The North American track l/w ratio average is more consistent with HBR_B3.2. As demonstrated, the HBR_B3 subgroups exhibit variable track metrics which overlap with multiple examples of individual ichnotaxa (i.e. *Carmelopodus*) from this time period around the world as well as *Grallator*.

Overall, HBR_B3 possesses multiple metrics and morphologies which cannot be collectively attributed to one ichnotaxon. The generally narrower <50° divarication angles, pronounced l/w ratios and mesaxony of HBR_B3.1 is more consistent with *Grallator* than HBR_B3.2 (e.g. SM.1976.2022.012-1). The weak <1.40 l/w ratios and wider >50° divarication angles, in combination with well-separated digits and the occasional presence of a ‘2 : 3 : 3’ phalangeal pad formula, of HBR_B3.2 tracks are more in line with *Carmelopodus* than *Grallator*—especially negative relief tracks from Lùb Score (e.g. SM.1976.2023.004-1). Despite this, the association and morphological similarity (i.e. gracile, tapering digits, elongated and narrow ungual marks, and overlapping mesaxony) of HBR_B3.2 with HBR_B3.1 leads us to consider the subgroup as mostly *Grallator*-like with the characteristics of *Carmelopodus*.

### 3.11. HBR_B4

**Figure 29.**
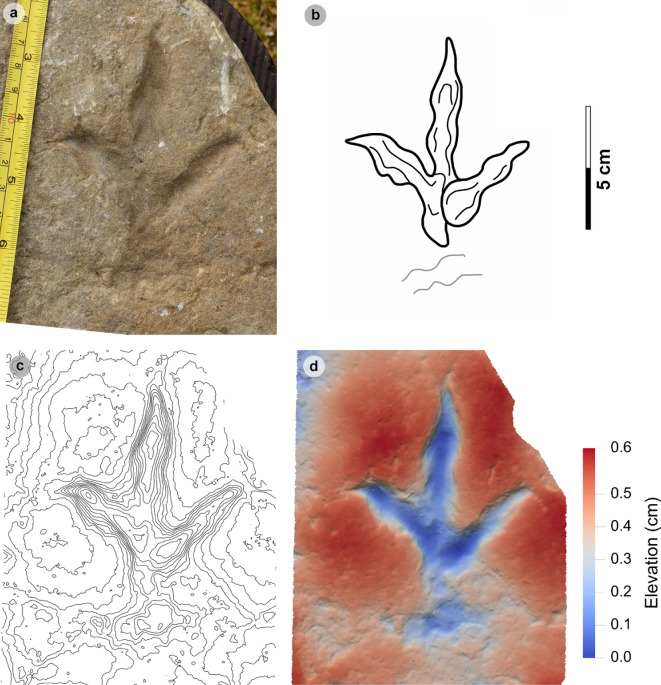
Photographic and digital representations of SM.1976.2009.002. (a) Photograph with scale, (b) outline, (c) 0.5 mm contour map and (d) DEM. As well as a ‘X : 2 : 2 : ?’ phalangeal pad formula based on oval-shaped concave pads, the track possesses a short, posteriorly oriented hallux and sharp, elongated ungual marks.

**Figure 30.**
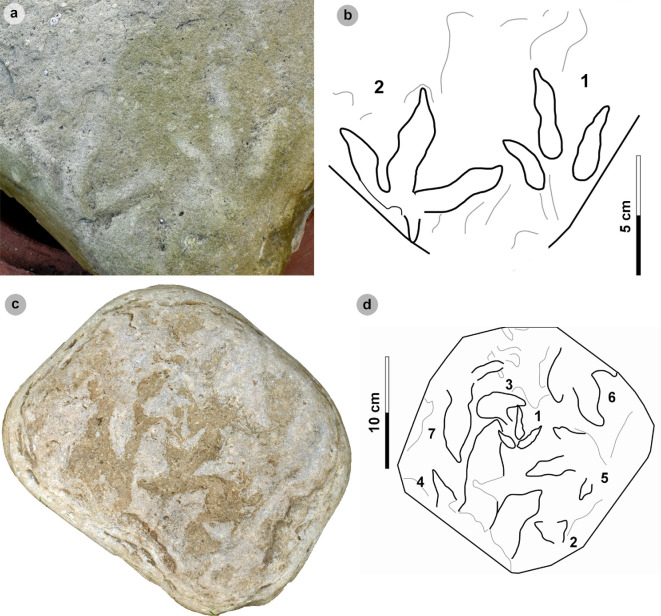
Photographs and outlines of surface worn HBR_B4 tracks. (a,b) SM.1976.2022.013-1−2 exhibit contrasting l/w ratios and divarication angles. Track 2 may also feature a worn hallux. (c,d) SM.1976.2022.007-1 is registered amongst numerous other tiny-sized tracks. Note the outline diagram covers the cropped area of a three-dimensional model.

**Material**: SM.1976.2009.002, SM.1976.2022.007-1, SM.1976.2022.01-1−2 (figures 29,30 and 30, table 15)

**Table 15.** Measurements of HBR_B4 tracks.

						digit length (DL)			DL ratios			divarication angles				
specimen	PG	L/R	L	W	L/W	II	III	IV	III/II	III/IV	III/L	II–III	III–IV	II–IV	te	M
2009.002	2	L	6.44	5.80	1.11	3.95	4.67	3.82	1.18	1.22	72.52	49.87	40.25	90.12	3.16	0.54
2022.007-1	1	R	3.85	3.61	1.07	2.33	2.86	2.58	1.23	1.11	74.29	39.97	38.36	78.33	1.83	0.51
2022.013-1	1	R	5.02	4.21	1.19	2.23	3.66	2.73	1.64	1.34	72.91	37.59	25.62	63.21	2.21	0.52
2022.013-2	1	R?	5.16	5.75	0.90	3.02	4.61	3.65	1.53	1.26	89.34	47.34	45.38	92.72	2.67	0.46
***average***	***1.25***	**—**	***5.12***	***4.84***	***1.07***	***2.88***	***3.95***	***3.20***	***1.39***	***1.23***	***77.26***	***43.69***	***37.40***	***81.10***	***2.47***	***0.51***

All lengths were measured in cm. The III/L ratio is expressed as a percentage. Divarication angles were measured in degrees. All track measurements were rounded to two decimal places.

#### 3.11.1. Diagnosis

Tracks are <10 cm length (tiny) with moderate mesaxony (approx. 0.50); digits ii–iv are often widely divergent (>60°) and can converge centrally under the posterior margin of digit iii. A posteriorly oriented, subtriangular hallux may be impressed.

#### 3.11.2. Description

Tiny, asymmetric, tridactyl footprint, 3.9−6.4 cm long and 3.6−5.8 cm wide, with relatively subequal l/w ratios (0.90−1.19) and moderate mesaxony (0.46−0.54). Digits are slender and taper distally, occasionally toward narrow, elongated, triangular ungual marks. On average, digit iii occupies 77.3% of the total track length. Digits ii and iv are subequal in length. The posterolateral margins of digits ii–iv can converge under digit iii. Occasionally, a posteriorly oriented subtriangular hallux is impressed. Digit ii–iv divarication angles range between 63.2° and 92.7°. The clearest tracks can feature a ‘X : 2 : 2 : ?’ phalangeal pad formula.

#### 3.11.3. HBR_B4 material description

Morphotype HBR_B4 is the smallest Hebridean morphotype described and currently known exclusively from Lùb Score. SM.1976.2009.002 is the only negative relief example of HBR_B4 to possess a short, reversed hallux and sharply defined, oval-shaped phalangeal pad margins (figure 29). The pads make up a ‘X : 2 : 2 : ?’ formula and are defined by equally sharp digit margins. Unlike HBR_B3, the lateral digits appear to converge directly below the posterior margin of digit iii while maintaining a wide digit ii−iv divarication angle (90.1°). The track occurs on a featureless surface composed of fine-grained sandstone similar to SM.1976.2022.12-1. The other three HBR_B4 tracks are surface worn and occur in surfaces of an uncorrelated horizon of poorly consolidated, fine-grained sandstone (figure 30). Although similar in size to one another, SM.1976.2022.013-1−2 possess subtly different metrics which occupy the extreme ranges of HBR_B4. Track 1 is subtly longer than wide (l/w = 1.19) and acutely divaricated between digits ii–iv (63.2°). In contrast, track 2 is subtly wider than long (l/w = 0.90) and subtly obtusely divaricated between digits ii–iv (92.7°). Track 2 furthermore may possess a posteriorly directed hallux which is absent in track 1 (figure 30a,b). SM.1976.2022.007-1 in contrast occurs among at least seven other tiny tracks bearing multiple directions—many incomplete and potentially represent slightly larger trackmakers (figure 30c,d). The track is characterized by similar metrics to SM.1976.2022.013-1, a weak l/w ratio (1.07) and moderate mesaxony (0.51). The HBR_B4 trackmaker was clearly present in the same palaeoenvironment as these slightly larger trackmakers in a roughly similar timeframe.

#### 3.11.4. HBR_B4 ichnotaxonomy

The wide digit ii–iv divarication angles (up to 92°), subequal lateral digit length, and short, posteriorly directed, subtriangular hallux of HBR_B4 are features often associated with bird-like ichnotaxa such as *Plesiornis* and *Trisauropodiscus* [83,84]. Note, however, that it is not necessarily the case that these tracks were made by true birds, as many small non-avian theropods had generalized bird-like feet [85]. Early Jurassic *Plesiornis pilulatus* tracks from Poland, like HBR_B4, are on average 4.6 cm long, divaricated by 61°−92° between digits ii and iv, and possess an average l/w ratio of 1.08 [84,86]. Despite its similarly gracile digits, unlike HBR_B4, *Plesiornis* possesses a deeply impressed digit iii metatarsophalangeal pad—a morphology commonly associated with avian tracks [84].

Early Jurassic *Trisauropodiscus* tracks from South Africa feature similar gracile digits and often poorly defined phalangeal pads like most HBR_B4 tracks [83]. Middle Jurassic examples of *Trisauropodiscus* from Morocco, similar to some HBR_B4 tracks, are wider than long (l/w ratios of 0.80−0.90), mesaxonic and obtusely divaricated >90° between digits ii and iv [87]. The lateral digits of the Moroccan tracks also convergent directly under the posterior margin of digit iii and above a reversed hallux. In the Middle Jurassic Cleveland basin in Yorkshire, equivalently or more widely divaricated and gracile digited morphotypes, including Bxiii and Bxiv [65], further resemble *Trisauropodiscus* and HBR_B4. Based on these similarities, HBR_B4 is most similar to *Trisauropodiscus*.

## 4. Discussion

### 4.1. Comparable assemblages

Prior to this study, the existing record of well-described tiny–medium theropod tracks on Skye was minimal [18,23–25,65]. Here, after a comprehensive survey of the existing Staffin Museum collection and further fieldwork, we describe a variety of tiny to medium-sized tridactyl track morphotypes from Valtos and Lùb Score. The tracks provide evidence of an abundance of small theropod trackmakers, which inhabited various localized palaeoenvironments within a broader fluviodeltaic and freshwater–brackish system in the Middle Jurassic on Skye. The scarcity of large tridactyl tracks (>30 cm track length), as commonly recorded elsewhere in the Great Estuarine Group [17,20–22], is notable.

In the Lealt Shale Formation, which underlies the Valtos Sandstone Formation, a single, isolated HBR_B3.1 track (BP3_13) was recorded at a track site (BP3 at Rubha nam Bràithrean) dominated by larger tridactyl footprints [21]. Elsewhere, an *ex situ* medium-sized tridactyl track (presently privately owned) was identified in the Duntulm Formation at An Corran—a track site dominated by large theropod tracks [17,88]. The track has been referred as possibly ornithopod by [16]. Indeed, we measured ornithopod-like metrics on this specimen, including a weak l/w ratio (1.27) and relatively wide divarication angle (63.1°). Despite this apparent affinity, the balance of evidence favours this specimen being a worn theropod track. The track possesses a pronounced mesaxony (0.62) and features faint slender digits (these have been varnished and appear broader than reality), which taper toward sharp, elongated ungual marks (the clearest is anterolaterally oriented on digit iv). Two medium-sized tridactyl tracks are further recorded at Prince Charles’s Point—another tracksite dominated by large theropod tracks [22]. Most Middle Jurassic assemblages in Europe with large numbers of theropod tracks are also mostly composed of large sized morphotypes, many attributed to *Megalosauripus* isp. [30,31].

In contrast with these assemblages, the Aalenian–Bathonian aged rocks of the Cleveland Basin (Yorkshire, northern England) currently feature at least 18 tridactyl morphotypes—many also tiny–medium sized. Each morphotype is differentiated by at least two unique characteristics [28, p. 201]. A review by [65] aimed to apply this morphotype classification to Hebridean tracks. Despite some ichnotaxonomic similarity in the Cleveland Basin, i.e. tracks attributed to *Eubrontes* and *Grallator* [28], we determined most Hebridean tracks do not correspond well with the Cleveland Basin morphotype descriptions. For example, Hebridean tracks originally classified under the Cleveland morphotype Bxi (e.g. GLAHM 114904, since reclassified to HBR_B3.2—see electronic supplementary material, figure S4) lack digit ii–iv divarication angles between ‘85°−100°’ and a strictly triangular heel, and possess a digit iii which constitutes >60% of the total track length—not ‘50–60%’ as per Cleveland Basin tracks described in [28, p. 212]. Hebridean tracks originally classed under Biv and Bv (reclassified here to HBR_B2.1 or HBR_B2.2) typically do not possess digits with clearly ‘rounded terminations’ or strictly divergent lateral digits (e.g. SM 1976.2002.007-1). The only similarly defined tridactyl morphotype represented in both basins is that attributed to *Megalosauripus* isp., i.e. morphotype Bxviii in the Cleveland Basin and HBR_B1.1 in the Sea of the Hebrides Basin. On Skye, given the abundance of large-sized theropod tracks at some *in situ* localities [17,22], it is unlikely that their relative absence at Valtos or Lùb Score is necessarily reflective of overall composition of the local dinosaur community. While Clark & Brett-Surman [25] suggest a size bias could be influenced by limited present-day coastal exposures of track-bearing sediments, we additionally consider the possibility of localized sorting between different palaeoenvironments and their respective proximity or suitability for small-theropod trackmakers.

Previous comparisons with the Cleveland morphotype series in [65] have led to suggestions that some ‘medium–large’ sized tracks from the Hebrides Basin may represent ornithopod trackmakers. This possibility acquires new resonance with the recent description of a fragmentary partial dinosaur specimen from the Kilmaluag Formation of the Inner Hebrides Basin, which although difficult to classify with certainty, might represent an ornithopod [13]. We are confident an ornithopod affinity is not supported for the tracks from the Valtos and Lùb Score localities under study here. For large-sized tracks such as SM 1976.2002.008a, which have some ornithopod-like characters, such as weak l/w ratios and mesaxony, we diagnose a theropod affinity due to sharp, elongated ungual marks, vague phalangeal pads and a clear indication of moderately slender, pronounced digits based on the track DEM. Similar large theropod tracks with these characters are reported throughout the Great Estuarine Group [17,20–22]. Such tracks may feature worn digit margins, which create the illusion of tracks having an ornithopod affinity, especially Hebridean morphotype HBR_B2.3. Any diagnoses through footprints must be cautiously undertaken with respect to numerous processes involved in shaping a track (*sensu* [89]).

North American tiny to medium-sized theropod tracks from Middle Jurassic sediments of the Bighorn Basin, Wyoming, were determined by [25] to be generally indistinguishable from the Valtos and Lùb Score assemblages. Indeed, although mostly larger (>15 cm in length), the Bighorn Basin tracks figured in [25,90,91] resemble HBR_B3.2 tracks present at Lùb Score. The tracks are characterized by gracile, well-separated digits (digit iv is on average longer than ii) like HBR_B3.2. An example grallatorid track described in [27, p. 248] from Sheep Mountain further resembles HBR_B3.1 with a narrow divarication angle and gracile digits tapering to sharp ungual marks.

### 4.2. Morphotype and assemblage comparison

**Figure 31.**
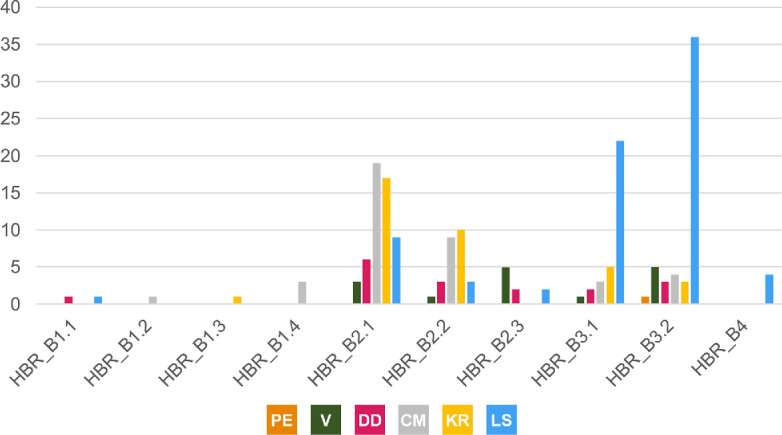
Morphotype subgroup abundance across Valtos and Lùb Score. Valtos is split into the following sublocalities: Port Earlish (PE), Valtos (V), Dun Dearg (DD), Carraig Mhòr (CM) and a rockfall north of Kilt Rock (KR). As tracks are found across Lùb Score (LS) and are not constrained to specific sublocalities as observed at Valtos, we represent the tracks under the entire locality. The abundance chart also includes tracks referred to each morphotype.

HBR_B2 is the most common morphotype in the Valtos assemblage and is characterized by small- to medium-sized eubrontid-like tracks (figure 31). HBR_B2.1 is considered most similar to *Eubrontes*. In the Lùb Score assemblage, HBR_B3 dominates and is characterized by tiny to small-sized grallatorid-like tracks (figure 31). HBR_B3.1 is considered most similar to *Grallator*. Previous descriptions of the Lùb Score tracksite assemblage of eubrontid and grallatorid tracks have suggested that despite variation in size, the tracks originate from a ‘monospecific ichnotaxon group of young and adult dinosaurs’ [24, p. 100]. This relationship is most clearly evidenced with reference to specimen SM 1976.2002.007 (figures 11, 21,25 and 25) which is composed of several associated smaller HBR_B3 tracks bearing a similar direction to an individual example of a larger HBR_B2.1 track.

It is challenging to determine whether these particular Skye tracks were made by two species (one larger and one smaller) or by different ontogenetic stages of the same trackmaker species. Studies on well-known theropod footprint assemblages outside Skye can provide some context. In North America, Early Jurassic *Grallator* and *Eubrontes* tracks from the Connecticut Valley were distinguished by hypothesized allometric changes (described under the ‘*Grallator-Eubrontes* plexus’) such as a reduction in mesaxony and increase in digit ii–iv divarication angle as tridactyl tracks across this grouping increased in size—from *Grallator* to *Eubrontes* [60,92]. This could be evidence for a monospecific trackmaker for these tracks (*sensu* [59]). The *Grallator-Eubrontes* plexus hypothesis, however, has been challenged by several studies [93–96]. Most critically, following a thorough statistical analysis of the Connecticut Valley tracks, Farlow [93] suggests that *Grallator* and *Eubrontes* were made by different trackmaking taxa.

**Figure 32.**
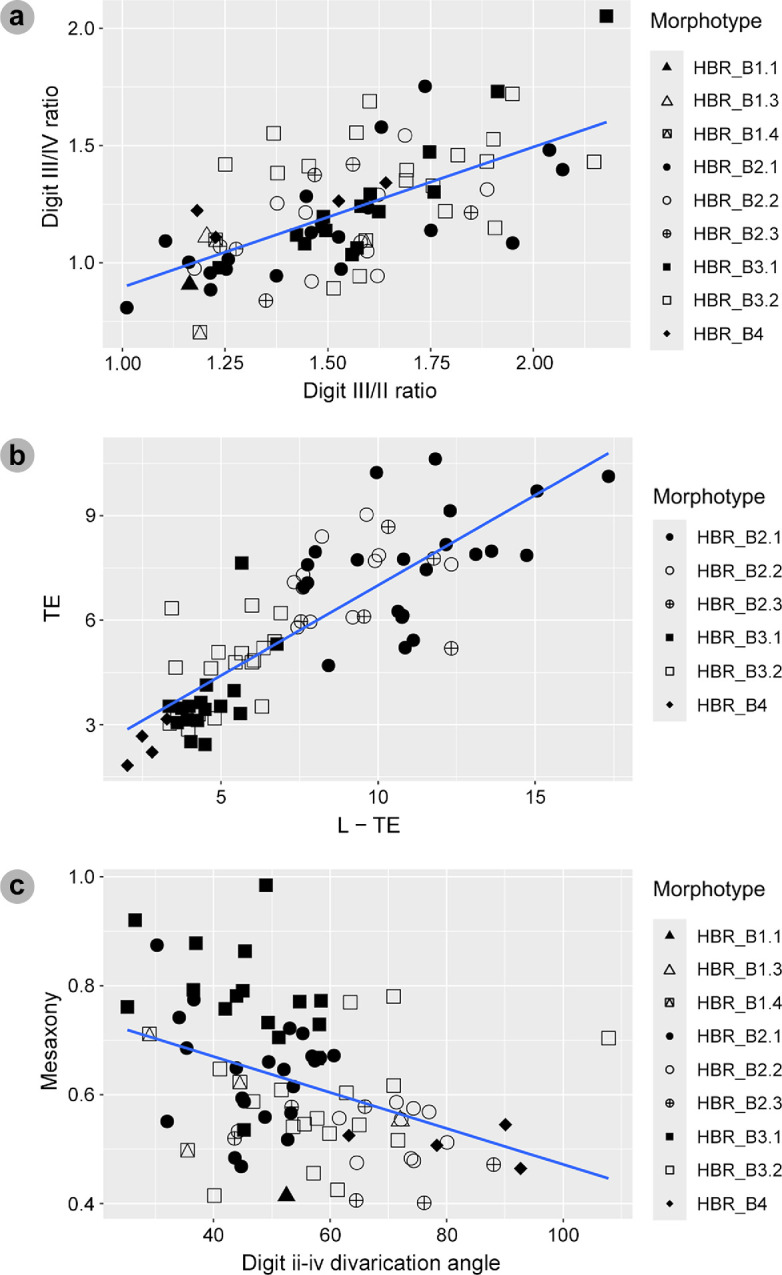
Scatterplots of track metrics. (a) Scatterplot of morphotype digit length ratios. In contrast to the hypothesized *Grallator-Eubrontes* ontogenetic single-trackmaker plexus [59], there are no clear allometric trends between HBR_B3 and HBR_B2 (i.e. grallatorid to eubrontid) as tracks of variable lengths are clustered randomly despite a positive correlation (Pearson correlation coefficient = 0.6249721). Note three HBR_B2.3 tracks are excluded due to insufficient measurements. (b) Scatterplot of morphotype mesaxony. Triangle elongation (TE) indicates the digit iii pronouncement when compared with the remaining track length, i.e. L-TE (*sensu* [92]). In [92], the hypothesis of allometric changes of a single trackmaker is indicated when TE declines in larger eubrontids from a sharper increase in smaller grallatorids. The Valtos/Lùb Score tracks instead show a broader ranged increase (Pearson correlation coefficient = 0.8166064). Note that all HBR_B1 subgroups are excluded. Three HBR_B2.3 tracks are excluded due to poorly defined digit margins. (c) Scatterplot of morphotype mesaxony and digit ii–iv divarication angle. Regardless of morphotype, mesaxony declines as divarication angle increases (Pearson correlation coefficient = −0.4078941). This likely explains why narrowly divaricated subgroups (i.e. HBR_B2.1 and 3.1) predominantly cluster separately from broadly divaricated subgroups (i.e. HBR_B2.2 and 3.2). Note that HBR_B1.2 and three HBR_B2.3 tracks are omitted due to the necessary measurements not being possible on the material in this sample.

For the Skye tracks, the digit ratios for the Valtos/Lùb Score tracks are considerably more varied between morphotypes and broadly overlap with one another (figure 32a). Furthermore, the increase in digit iii pronouncement (TE) does not clearly reduce in larger morphotypes (HBR_B2) from a sharper increase in smaller morphotypes (HBR_B3), as seen in [92], when compared with the remaining track length (L–TE) (figure 32b). For the Hebridean morphotypes, TE is likely a function of digit ii–iv divarication angles on the position of the width line (from which TE is measured). The mesaxony reduces as the lateral digits diverge further apart regardless of morphotype (figure 32c). This observed pattern is unsurprising given the way these measurements are defined in that they are interlinked and interdependent in context of the whole track.

Therefore, based on comparisons between the digit length ratios, mesaxony and divarication angles of HBR_B2 and HBR_B3, we do not observe consistent allometric changes between grallatorid and eubrontid tracks within the Hebrides assemblage. As such, ontogenetic variations within a single trackmaking taxon do not appear to be the strongest explanation for these differently sized tracks overall. The variation in morphotype morphology and metrics within the Valtos and Lùb Score assemblages is most likely influenced by a combination of variables, such as substrate conditions and locomotion style (behaviour) [60,89,97,98] rather than a single explanatory factor like ontogenetic changes in one trackmaker.

We do, however, observe some potential association between small (i.e. HBR_B3) and large (i.e. HBR_B2) bodied trackmakers on exceptional track-bearing surfaces like SM.1976.2002.007 (figures 11, 21,25 and 25). In this particular case, this association might represent juveniles and adults of the same taxon [24]. However, this story from track associations seems to conflict with the patterns evidenced from track measurements (figure 32). While we cannot fully rule out the possibility that some Skye footprints were made by juveniles (and will further consider the implications of SM.1976.2002.007 in the following section), the balance of the evidence favours multiple trackmaking taxa of different body sizes within the local dinosaur assemblage *overall*. With that said, there may be localized instances of juvenile tracks, and these tracks might overlap in metrics with the tracks of smaller-bodied adult trackmakers, and thus would be classified into the same morphotype category.

### 4.3. Trackmaker behaviour in context to palaeoenvironment

**Figure 33.**
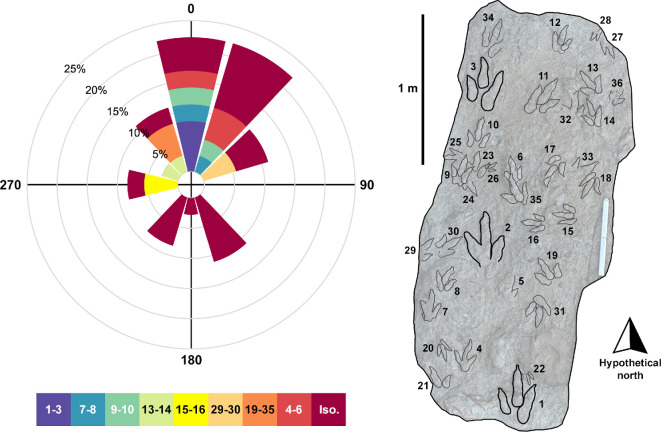
Windrose diagram of VA10 track bearings. Track bearings were determined digitally using a hypothetical north as a reference point as VA10 is *ex situ*. Trackways and track associations are colour coded on the windrose. Note that the ‘Iso.’ label corresponds to isolated tracks. Overall, tracks are observed bearing multiple directions.

Behavioural interpretations from either Skye assemblage are limited to track-bearing slabs with multiple tracks distributed across relatively small surfaces areas. Some of the most interpretable specimens from Valtos are VA09 and VA10 (figures 14,17 and 17)—composed of medium- to coarse-grained sandy limestones which represent ‘brackish transgressions of a lagoon shoreline’ [36, p. 127]. VA10 comprises a moderately dinoturbated surface (*sensu* [99]), with at least 36 tracks representing three morphotypes bearing multiple directions across an approximately 3.1 m^2^ area (figure 33). VA09 is composed of 11 tracks representing two morphotypes bearing multiple directions over a smaller area (approx. 0.7 m^2^). Past studies have interpreted gregarious behaviours from structurally spaced and parallel trackways [24,100–103]. Such patterns, however, are not evident from the Valtos blocks which feature more disorganized track directions (figure 33). These unpreferred directions suggest the Valtos trackmakers were unrestricted by any immediate local topography, i.e. shoreline position, and traversed independently rather than in a gregarious group [104,105].

Some VA10 tracks appear associated over relatively short sequential paces or strides, e.g. VA10-7−8, 9−10 and 15−16. Similar multi-directional bearing tracks are represented in desiccated surfaces, e.g. SM.1976.2002.008b, SM.1976.2021.001 and VA14 (figures 10i–k–34 and 34). These can be overprinted and vary in margin sharpness—collectively evidencing some time-averaging across these blocks. In SM.1976.2021.001, tracks 1a-1 and 1b-1 possess sharper margins than track 1b-2—which features more pronounced desiccation cracks running across the track. Similar tracks are noted in VA14, with track 4 overprinting track 7. In VA10, track 9 overprints tracks 23−25 (figure 14).

**Figure 34.**
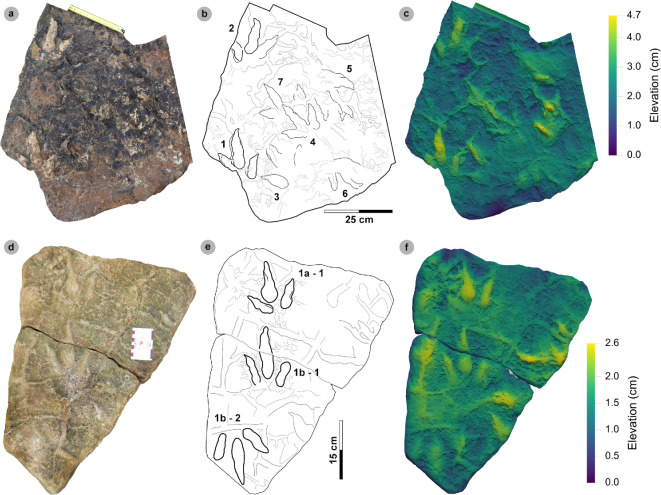
Digital and photographic representations of associated tracks on desiccated surfaces. Photograph/textured orthomosaic, outline with tracks numbered and DEM of (a–c) VA14 and (d–f) SM.1976.2021.001. On VA14, track 4 clearly overprints track 7 but features weaker digit margins with greater surface desiccation compared to associated tracks 1−2 with sharper margins. On SM.1976.2021.001, track 1b-2 contrasts 1a-1 and 1b-1 with weakly defined, broader and rounder digit margins and desiccation cracks cutting across it.

The short paces or strides and variation in track bearings may reflect a form of milling behaviour [104]. Extant shorebirds similarly pace short distances and increase their rate of turning while foraging and feeding in prey rich areas [106]. Early Cretaceous shorebird tracks from Korea exhibit these characteristics, with additional peck marks in association with invertebrate trace fossils that likely signify feeding behaviour [107]. Invertebrate trace fossils and equivalent feeding marks, however, are absent from track-bearing surfaces including VA10. Despite this, given the tidal influence of these brackish lagoons [34], the Valtos trackmakers may have foraged during low tides for non-burrowing fauna. Indeed, the density of these time-averaged tracks could have been influenced by the area of accessible shoreline imposed by tidal cycles as observed on the feeding habits of extant shorebirds [108,109].

Although dominated by morphotype HBR_B2, the presence of multiple morphotypes in VA09 and VA10 further indicates trackmakers of differing sizes coexisted. In VA10, HBR_B1.4 tracks occur in a trackway with consistently large pace lengths (1.05−1.06 m) associated with a faster 1.70−1.88 m s^−1^ (6.12−6.77 km h^−1^ = 3.80–4.21 mph) velocity and walking gait (1.54) than implied by the paces or strides of smaller trackmakers. The HBR_B1.4 trackway suggest independent behaviour from the HBR_B2 trackmakers—possibly passing through (figures 8 and 33). Though represented by two isolated tracks on VA10, HBR_B3 trackmakers may have also roamed independently as the tracks also bear multiple directions.

In contrast to the Valtos slabs, multiple morphotype HBR_B3 tracks are often found together in track-bearing surfaces from Lùb Score. The most notable, SM 1976.2002.007, contains 28 tracks attributed to this morphotype group with a single medium-sized morphotype HBR_B2.1 track. As discussed in [24], the shared southwesterly direction of both track morphotypes and lack of overprinting between the footprints may represent possible post-hatchling care and parallel directed herding behaviour. Parallel directed gregariousness has been widely documented in sauropod and ornithopod trackmakers in addition to theropods [101,103,110,111].

When considered at a subgroup level, the effects of substrates on the morphotypes between the Valtos Sandstone and Kilmaluag Formations are broadly independent from our classification (except for HBR_B1.2 and HBR_2.3). The Hebridean morphotypes occur in a variety of substrates, i.e. rippled or desiccation cracked. The overarching difference between morphotypes HBR_B2 and HBR_B3 is in their relative abundances within the Valtos Sandstone and the Kilmaluag Formations. A greater abundance of smaller morphotypes (i.e. HBR_B3) is present in the freshwater-influenced Kilmaluag Formation in contrast to the larger morphotypes (i.e. HBR_B2) dominating the freshwater–brackish and fluviodeltaic-influenced Valtos Sandstone Formation. In [25], it was speculated that the lagoonal palaeoenvironments may have provided the Skye and comparable North American Middle Jurassic trackmakers with specific exploitable palaeoecological niches, i.e. feeding on nearshore aquatic prey (i.e. fish, invertebrates). The ability to undertake this ecological strategy may in part be due to the proximity of these more marginal coastal environments to well-established terrestrial ecosystems—indicated by plant debris (although generally absent from track-bearing horizons). The closed-lagoonal setting at Lùb Score may have provided smaller trackmakers with more favourable ecological niches, such as greater protection from predators or more abundant favourable food sources. An alternative hypothesis, which we explore here, considers proximity to suitable nesting habitat.

Dinosaurs are known to have nested in a variety of settings, including nearshore ones. Evidence of dinosaurs nesting on a tidal flat, in a lagoonal depositional system, was recorded in a Late Cretaceous marl with plant debris and euryhaline ostracods in Spain [112]. Similarly aged eggs attributed to small theropods were reported in emerged beach ridges of a barrier island, lagoonal system, also in Spain [113]. Eggshells and nests have yet to be recorded in the Great Estuarine Group, but this is not surprising given how rare these delicate fossils are globally, and how they must be preserved in suitable sedimentological and geochemical settings [114]. With that said, tiny tracks such as GLAHM 114913-1 and 2, which are ≤2 cm long and accompanied by a larger 8 cm track (GLAHM 114913), as originally described by [24], may have formed part of an adult–juvenile (and may be even parent–offspring) grouping, which then might imply that nesting sites existed nearby. SM.1976.2022.007-1, a 3.85 cm long track (reflective of a tiny trackmaker with a hip height around approx. 15 cm), similarly occurs among other larger tiny to small-sized tracks.

Without direct evidence to support the suitability of Skye’s lagoonal palaeoenvironments as potential nesting grounds (i.e. eggshell or nest remains) or habitat for smaller trackmakers, the reasons for their abundance in the Lùb Score assemblage remain an open question. Track-bearing surfaces with multiple tracks currently offer the most interpretation on palaeoenvironment and trackmaker behaviour.

### 4.4. Possible trackmaker identification

Theropod body fossils from the Great Estuarine Group are rare and mostly consist of teeth [9,10,12]. It was concluded in [12] that it was likely that tooth specimens NMS G.2018.17.1 and GLAHM 125390a originated from a large non-maniraptoriform theropod akin to a ceratosaur, megalosauroid or allosauroid.

Morphotype HBR_B1.1 possesses a similarly weak l/w ratio (1.31) and mesaxony (0.41), and moderate digit ii–iv divarication angle (52.5°) to tracks attributed to large carnivorous theropods including megalosaurids [17,22,30,31,67,70]. Morphological similarities include narrow, tapering ungual marks and broad, padded digits. Based on the reported morphotype track length (43.9 cm), the trackmaker possessed a hip height of approximately 1.76 m. Based on the discussion on potential trackmakers for <50 cm long tracks in [115], we suggest HBR_B1.1 was registered by a theropod with a body length of approximately 5 m. In Britain, theropods of this general size include *Megalosaurus bucklandii* [116]—a widely distributed Bathonian English megalosaurid estimated to have grown up to approximately 6 m long. A similar large English theropod, *Duriavenator hesperis*, grew up to approximately 7 m [117] and may have registered tracks of similar length to HBR_B1.1. Additional megalosaurids including *Cruxicheiros newmanorum* [118] and *Magnosaurus nethercombensis* [119] are presently represented by subadult specimens which could have grown to larger body sizes and registered tracks of similar length to HBR_B1.1.

Despite their smaller <40 cm track lengths, morphotypes HBR_B1.3 and HBR_B1.4 are metrically, and morphologically, similar to morphotype HBR_B1.1. Although also more widely divaricated, HBR_B1.3 is similar to HBR_B1.1, with weak l/w ratios (1.29) and a digit iii that is 60.1% of the track length. The similarities of morphotype HBR_B1.4 with *Therangospodus* are notable, as this ichnotaxon is often found associated with ichnotaxa referred to megalosaurids (i.e. *Megalosauripus*) [76]. Both morphotypes could hypothetically represent a juvenile megalosaurid trackmaker of a similar species represented by morphotype HBR_B1.1—with an estimated hip height between 1.27 and 1.43 m. However, a lack of tracks/trackways prevents allometric comparison to increase certainty. Regionally appropriate trackmakers with equivalent hip heights include *Eustreptospondylus oxoniensis* [120].

The tiny to medium-sized morphotypes were likely registered by theropods with estimated hip heights between 5 and 100 cm. The morphological variation collectively exhibited by these morphotypes may imply that multiple trackmaking species of this size range existed on Skye. While this range could encompass juvenile megalosaurids, we also consider smaller theropods such as *Proceratosaurus bradleyi*—a 3 m long Middle Jurassic proceratosaurian theropod from England [121]. This theropod may have been capable of registering tracks approximately 20 cm in length. Body fossils of other small theropod taxa are also poorly recorded from English Middle Jurassic deposits and are mostly known from teeth [122]. On Skye, a middle caudal vertebra from the Valtos Sandstone Formation [10] was determined to likely originate from a basal coelurosaur, comparable to the 2 m long *Coelurus*—a small theropod from Late Jurassic deposits in North America [123]. A fragmentary tooth from the Valtos Sandstone Formation (NMS G.2018.17.2) bears affinities of ‘a neotheropod theropod other than a member of Abelisauridae, Megalosauria and Maniraptoriformes or possibly a ceratosaur closely related to Noasauridae’ [12, p. 14]. Cladistic analysis further suggested a dromaeosaurid identification [12], which would be consistent with the identification of maniraptoran teeth from English Bathonian sediments by [122]. Following the evidence of at least two theropod species present in the Great Estuarine Group based on teeth [12] and given the variety of species discovered in Britain, it is likely that multiple theropod species existed in the Sea of the Hebrides basin. This hypothesis, however, remains speculative in the absence of more definitive body fossils. At present, based on local body fossil material, we suggest the tiny to medium-sized track morphotypes were impressed by small-bodied members of a basal coelurosaur or non-coelurosaurian group (e.g. Ceratosauria, Megalosauroidea, Allosauroidea).

## 5. Conclusion

In the Bathonian aged Valtos Sandstone Formation at Valtos and Kilmaluag Formation of Lùb Score on the Isle of Skye, Scotland, we describe and classify 185 dinosaur tracks into four morphotypes within a new Hebridean series. We establish that the smallest trackmakers, represented by the grallatorid morphotype HBR_B3, occurred more abundantly in the low salinity, closed lagoons at Lùb Score than larger trackmakers, represented by the eubrontid morphotype HBR_B2, in the fluviodeltaic shore-margin complex at Valtos. This difference may be attributed to the proximity or function of local palaeoenvironments for specific trackmakers. At Valtos, short paces and strides of larger trackmakers on track-rich surfaces may indicate specific behaviours such as foraging. At Lùb Score, parallel-directed tracks likely imply gregarious behaviour between different morphotype trackmakers—highlighting potential post-hatchling care if they were made by juveniles and adults of the same species instead of adults of a larger and smaller species. The Valtos and Lùb Score tracks may represent several theropod trackmaker groups, possibly a large megalosaurid and multiple smaller-bodied basal coelurosaurian or non-coelurosaurian theropods (e.g. Ceratosauria, Megalosauroidea, Allosauroidea). Overall, the dominance of tiny to medium-sized tracks across multiple beds and formations establishes the prominence of small theropod trackmakers on Skye during the Middle Jurassic.

## Data Availability

Supplementary data include a series of photogrammetric models (and associated image sets) of representative dinosaur tracks used to characterize a new Hebridean morphotype series. The tracks originate from Valtos and Lùb Score on the Isle of Skye, Scotland. Also included are a set of .csv files used to make scatterplots and a windrose diagram for the VA10 multi-track surface. These data are hosted on Zenodo and accessible at: [124]. Supplementary material is available online [125].
